# Identification and Characterization of the Host Protein DNAJC14 as a Broadly Active Flavivirus Replication Modulator

**DOI:** 10.1371/journal.ppat.1001255

**Published:** 2011-01-13

**Authors:** Zhigang Yi, Lindsey Sperzel, Cindy Nürnberger, Peter J. Bredenbeek, Kirk J. Lubick, Sonja M. Best, Cristina T. Stoyanov, Lok Man J. Law, Zhenghong Yuan, Charles M. Rice, Margaret R. MacDonald

**Affiliations:** 1 Laboratory of Virology and Infectious Disease, The Rockefeller University, New York, New York, United States of America; 2 Key Laboratory of Medical Molecular Virology, Shanghai Medical College, Fudan University, Shanghai, China; 3 Department of Medical Microbiology, Leiden University Medical Center, Leiden, The Netherlands; 4 Laboratory of Virology, National Institute of Allergy and Infectious Diseases, National Institutes of Health, Rocky Mountain Laboratories, Hamilton, Montana, United States of America; Washington University School of Medicine, United States of America

## Abstract

Viruses in the *Flavivirus* genus of the *Flaviviridae* family are arthropod-transmitted and contribute to staggering numbers of human infections and significant deaths annually across the globe. To identify cellular factors with antiviral activity against flaviviruses, we screened a cDNA library using an iterative approach. We identified a mammalian Hsp40 chaperone protein (DNAJC14) that when overexpressed was able to mediate protection from yellow fever virus (YFV)-induced cell death. Further studies revealed that DNAJC14 inhibits YFV at the step of viral RNA replication. Since replication of bovine viral diarrhea virus (BVDV), a member of the related *Pestivirus* genus, is also known to be modulated by DNAJC14, we tested the effect of this host factor on diverse *Flaviviridae* family members. Flaviviruses, including the pathogenic Asibi strain of YFV, Kunjin, and tick-borne Langat virus, as well as a *Hepacivirus*, hepatitis C virus (HCV), all were inhibited by overexpression of DNAJC14. Mutagenesis showed that both the J-domain and the C-terminal domain, which mediates self-interaction, are required for anti-YFV activity. We found that DNAJC14 does not block YFV nor HCV NS2-3 cleavage, and using non-inhibitory mutants demonstrate that DNAJC14 is recruited to YFV replication complexes. Immunofluorescence analysis demonstrated that endogenous DNAJC14 rearranges during infection and is found in replication complexes identified by dsRNA staining. Interestingly, silencing of endogenous DNAJC14 results in impaired YFV replication suggesting a requirement for DNAJC14 in YFV replication complex assembly. Finally, the antiviral activity of overexpressed DNAJC14 occurs in a time- and dose-dependent manner. DNAJC14 overexpression may disrupt the proper stoichiometry resulting in inhibition, which can be overcome upon restoration of the optimal ratios due to the accumulation of viral nonstructural proteins. Our findings, together with previously published work, suggest that the members of the *Flaviviridae* family have evolved in unique and important ways to interact with this host Hsp40 chaperone molecule.

## Author Summary

Viruses in the *Flavivirus* genus are transmitted by arthropods and cause significant disease burden across the globe. We undertook a screening approach to select for and identify host factors that provide resistance to death caused by infection with the mosquito-transmitted *Flavivirus*, yellow fever virus (YFV). We identified the host factor DNAJC14, an Hsp40 chaperone protein family member, as able to inhibit replication of the vaccine strain of YFV, and the virulent parental Asibi strain. We found that DNAJC14 also inhibits several other members of the *Flavivirus* genus, including Kunjin and the tick-borne Langat virus. Moreover, the *Hepacivirus* hepatitis C virus is also inhibited, suggesting a role for DNAJC14 in modulating the replication of all three genera of the *Flaviviridae* family. By probing the mechanism of the YFV inhibitory process, we determined that DNAJC14 inhibits at a post entry step, and most likely prevents the formation of functional replication complexes. We determined that DNAJC14 is required for YFV replication and that expression of inappropriately high levels of this protein results in a disruption of a process critical for viral RNA replication. Understanding how host factors inhibit or contribute to *Flavivirus* replication steps may identify new targets for antiviral drug development.

## Introduction

The *Flavivirus*, *Pestivirus* and *Hepacivirus* genera of the *Flaviviridae* family each include important human and/or animal pathogens [1]. A major human pathogen hepatitis C virus (HCV) is a member of the *Hepacivirus* genus, while *Pestiviruses* bovine viral diarrhea (BVDV), border disease and classical swine fever viruses each have significant economic consequences in the livestock industry. Within the *Flaviviridae* family, members of the *Flavivirus* genus, which includes over 50 viral species, have perhaps the most significant impact on human health [2], [3]. Viruses in this genus, including yellow fever (YF), dengue (DEN), West Nile (WN), Japanese encephalitis (JE) and tick-borne encephalitis (TBE) viruses, contribute to staggering numbers of human infections and significant death rates across the globe. Viruses in this genus are usually transmitted via arthropod vectors and as such human infection depends on climate and geographical factors affecting the ranges of the transmitting arthropod and the likelihood of arthropod-human contact. Rising global temperatures, increased human population densities, human movement and increased dispersal of ticks and mosquitoes have contributed to increased numbers of epidemics in new geographical locations; this trend is likely to continue.

While successful vaccines have been developed for prevention of YFV, JEV and TBEV infection, none are available for other pathogenic flaviviruses. Efforts to create and implement such vaccines have been hampered by the presence of multiple serotypes (DEN), the large geographical areas involved, and the sporadic nature of infection [4], [5]. Even for vaccine-preventable flavivirus infections, the cost associated with immunizing all at-risk people is enormous. Moreover, there are currently no drugs available for the specific treatment of any flaviviral disease. While several viral proteins are attractive targets for the development of small molecule inhibitors, the potential for rapid evolution of the flavivirus RNA genome suggests that resistance may be a significant problem. Disrupting critical interactions of viral proteins with host factors, or inducing expression of host proteins able to inhibit viral replication, are alternative approaches to developing effective anti-flaviviral therapies, and may limit the emergence of escape mutants. Unfortunately our understanding of host factor involvement in promoting or inhibiting flaviviral replication remains incomplete.

Members of the *Flavivirus* genus share a common genome organization and replication strategy [1]. After virion entry and fusion of the viral and host membranes within the endosome, the ∼11,000 nt viral positive sense genomic RNA is translated in association with host cell membranes to form a single polyprotein which is co- and post-translationally cleaved by both host and viral proteases. The structural proteins, C, prM and E, are located in the N-terminal region of the polyprotein, followed by the nonstructural proteins, NS1, NS2A, NS2B, NS3, NS4A, NS4B and NS5. After appropriate cleavage and assembly of replication complexes, the genomic RNA is replicated by NS5, the viral RNA-dependent RNA polymerase, in association with other viral nonstructural and host proteins to generate new progeny genomes. Virion morphogenesis then follows via encapsidation and budding into the ER lumen. Virions mature during transport through the secretory pathway and are released into the extracellular milieu via exocytosis.

Given the common replication strategy of the *Flavivirus* genus members, different species may exploit or be susceptible to many of the same host factors or environmental conditions. We hypothesized that identification of a cellular antiviral factor with activity against one flavivirus species may provide information on targets for broad-spectrum therapeutics. In order to identify antiviral cellular factors active against YFV, we conducted an iterative screen of a cDNA library from interferon-α treated cells. We identified DNAJC14, an Hsp40 family member, as able to mediate protection from YFV-induced cell death. Further studies demonstrate that DNAJC14 is recruited to YFV replication complexes and that its overexpression inhibits viral RNA accumulation. The C-terminus of DNAJC14, which mediates its multimerization, is required for antiviral activity. Furthermore, we found that silencing of endogenous DNAJC14 inhibits YFV replication. Overall our findings suggest that DNAJC14 plays an important role in regulating YFV replication complex assembly.

## Materials and Methods

### Plasmids

Retroviral vectors pV1, pV1-GFP, pTrip-EGFP and pTrip-TagRFP have been described [6], [7], [8], [9]. Derivatives were generated using standard methods; all polymerase chain reaction (PCR) generated sequences were verified by sequencing and primer sequences are available upon request. Derivatives of pV1 were constructed to express human DNAJC14 (hDNAJC14) and mutants, each containing a carboxyl-terminal myc tag; the myc tag (EQKLISEEDL) was introduced during PCR by inclusion of myc-encoding nucleotide sequences in the antisense primers. DNAJC14- or truncation mutant-encoding DNA was amplified, using the Expand Long Template PCR System (Roche), from hDNAJC14 cDNA plasmid (Open Biosystems), and after digestion with SfiI was cloned into similarly digested pV1. Plasmid pV1-hDNAJC14-FL encodes full-length hDNAJC14 (amino acids 1–702) with a carboxyl-terminal myc tag. The human N-terminal truncation mutant (NT1) corresponding to the truncated hamster cDNA isolated in the screen starts from the amino acid corresponding to residue 305 of hDNAJC14, and was designed to have the identical amino terminus as the hamster sequence (_305_M**V**QFLSQS—); the corresponding human wildtype sequence has a phenylalanine residue at position 306. Additional mutants generated include NT2: _250_AGFWWLIE—; NT3: _291_MGVWTGRL—; NT4: _320_FTRFLKLL—; NT5: _349_LVGLGDRL— and as necessary contained an extra methionine residue for translation initiation. C-terminal truncated mutants end at the following amino acids prior to the myc epitope tag: CT1: —HISFGSRI_625_; CT2: —DLKEAMNT_534_; CT3: —EEVARLLT_433_; CT4: —RFLVGLGD_354_; CT5: —AEELCQLG_248_. The NT5CT1 mutant contains both the NT5 and CT1 truncations. Point mutations were introduced using site directed mutagenesis with the appropriate oligos by standard techniques.

Plasmids pTrip-EGFP-hDNAJC14-NT5 and pTrip-RFP-hDNAJC14-FL or pTrip-RFP-hDNAJC14-NT1 were generated by PCR amplification of the NT5, full-length (FL) or NT1 hDNAJC14 sequences. After BsrG1 and XhoI digestion the sequences were ligated into similarly digested pTRIP-EGFP or pTRIP-TagRFP. These plasmids express hDNAJC14 (or mutants) fused in frame to the C-terminus of EGFP or RFP. To generate a doxycycline-inducible cell line expressing hDNAJC14-NT5, sequences encoding NT5 were generated by PCR and were inserted into pcDNA4/TO/myc-His B (Invitrogen) via HindIII and XbaI digestion to generate pcDNA4/TO/hDNAJC14-NT5. Plasmid pTrip-RFP-hNZAP was generated by cloning DNA encoding amino acids 1 to 252 of human zinc-finger antiviral protein [10], generated by PCR, as an in frame fusion with the carboxyl terminus of RFP in the pTrip-TagRFP vector.

Plasmids pFlag-HCV-NS2-3-WT or pFlag-HCV-NS2-3-H171A were described previously [11] and express Flag-tagged HCV NS2-3_181_ or the inactive H171A mutant form of the NS2 protease, respectively. Plasmids pFlag-YF-NS2-3(181)-WT or pFlag-YF-NS2-3(181)-S138A were generated by amplification of YFV NS2-3 fragments from plasmids pACNR-YF17D [12] and pET-BS(+)/Sig2A-5_356_-R2107/2506E, S1622A [13], respectively, and cloning the KpnI/XhoI digested products into similarly digested pcDNA3.1. The sense primer contained appropriate sequences to encode a Flag epitope tag on the N-termini of the respective proteins. The plasmids express Flag-tagged YFV NS2-3_181_ or the inactive S138A form of the NS3 protease, respectively.

Plasmids pACNR-FLYF17Dx [12] and pACNR-FLYF17Da [14] contain the sequences of YFV 17D downstream of the SP6 promoter with XhoI and AflII linearization sites, respectively. Plasmid pACNR-FLYF-Asibi (the details of which will be described in another paper) contains the sequences of YFV Asibi downstream of the SP6 promoter.

Plasmid pYF17D(5′C25Venus2AUbi) was constructed by inserting Venus, a variant of yellow fluorescent protein (YFP), into the YFV 17D open reading frame (ORF) using standard molecular techniques. All generated PCR products and plasmids were verified by restriction digests and by sequencing (primers available upon request). First, the Venus cassette was amplified from plasmid Venus/pCS2 (kindly provided by Dr. Atsushi Miyawaki) and cloned in frame after the first 25 amino acids of the YFV Capsid in pNEB193/YF5′ [15] using the SacI and AgeI sites. Next, the foot-and-mouth disease virus (FMDV) 2A peptide, which mediates cleavage following its own carboxy-terminus, and a ubiquitin (Ubi) monomer were amplified from pTM3-HCV-Ubi-NS5B (C. Lin and C.M. Rice, unpublished) and inserted downstream of Venus in pNEB193/YF5′ by assembly PCR. YFV 17D amino acids 1–514, containing silent mutations in sequences encoding the first 25 amino acids to avoid recombination, were similarly assembled downstream of FMDV 2A in pNEB193/YF5′. Finally, to generate pYF17D(5′C25Venus2AUbi), Venus and YFV 17D sequences were removed from pNEB193/YF5′ using SrfI and NsiI and cloned into pCC1-YF17D [16], which contains the entire YFV genome. Thus in pYF17D(5′C25Venus2AUbi), the Venus/2AUbi cassette is inserted in frame after the first 25 amino acids of Capsid, followed by the complete YFV polyprotein. The presence of FMDV 2A and Ubi downstream of Venus ensures complete cleavage from the YFV polyprotein, thereby ensuring the authentic YFV amino terminus. This strategy has the potential advantage of allowing expression of foreign inserts without disrupting the YFV 17D polyprotein, which may have unpredictable deleterious effects on replication.

YFV replicon plasmids pYF-R.luc2A-RP and pYF-luc-IRES-RP-ΔDD [17] were kindly provided by Richard J. Kuhn (Purdue University). Plasmid pYF-R.luc2A-RP-ΔDD (expressing a polymerase defective YFV luciferase-expressing replicon) was constructed by swapping the NdeI/XhoI fragment from pYF-luc-IRES-RP-ΔDD into pYF-R.luc2A-RP.

### Cell lines

All cell lines were maintained at 37°C in humidified chambers containing 5% CO_2_. SW13 (human adrenal carcinoma) cells were cultured in Minimum Essential Medium (MEM) Alpha Medium (MEMa, Invitrogen) supplemented with 10% fetal bovine serum (FBS, Invitrogen). Huh7.5 cells [18] were cultured in Dulbecco's Modified Eagle Medium (DMEM, Invitrogen) supplemented with nonessential amino acids (Invitrogen) and 10% FBS. HEK293T and Vero cells were cultured in DMEM supplemented with 10% FBS. T-REx-293-LacZ cells inducibly expressing myc-tagged LacZ were previously described [19]. T-REx-293-NT5 cells inducibly expressing hDNAJC14-NT5 were obtained by transfection of T-REx-293 cells with pcDNA4/TO/hDNAJC14-NT5 and selection in medium containing zeocin. The selected bulk population was then cultured in DMEM supplemented with 10% FBS, 5 µg/ml blasticidin, and 0.5 mg/ml zeocin. For induction, doxycycline was added to a final concentration of 1 µg/ml. BHK-J cells, a previously described [20] line of BHK-21 hamster kidney cells were cultured in MEM supplemented with 7.5% FBS and BHK/NZAP-Zeo cells [21] were maintained in the same medium with the addition of 200 µg/ml zeocin.

### Antibodies

Anti-Myc mouse monoclonal antibody 9E10 (ATCC CRL1792 hybridoma) was used in Western and immunofluorescence or immunoprecipitation at 2.5 and 16 µg/ml, respectively. Anti-Flag antibody (Sigma M2 mouse monoclonal) was used in Western analysis at 1∶1000 dilution. Yellow fever NS3 rabbit polyclonal antiserum was previously described [22] and utilized at 1∶5000 dilution for Western analysis, and 1∶500 for immunofluorescence. Rabbit polyclonal anti-GFP antiserum was generated as described [23] and utilized at 1∶20,000 dilution in Western and 1∶1000 in immunoprecipitation. Mouse monoclonal anti-calnexin antibody (BD Biosciences, 610523) was used in Western and immunofluorescence at 1∶250 and 1∶50 dilution, respectively. Mouse monoclonal anti-actin (Sigma, A5441) antibodies were utilized in Western analyses at 1∶5000 dilution. Rabbit polyclonal anti-DNAJC14 antibody (Sigma, HPA017653) was used in Western and immunofluorescence at 1∶2000 and 1∶200 dilution, respectively. Mouse monoclonal anti-double stranded RNA (dsRNA) J2 antibody (English & Scientific Consulting, Bt. Szirák, Hungary), kindly provided by Dr. Elena Frolova (University of Alabama at Birmingham), was used at 1∶200 dilution. Alexa Fluor 488 donkey anti-mouse IgG (A-212020) and Alexa Fluor 594 goat anti-rabbit IgG (A-11012, Invitrogen) were utilized in immunofluorescence at 1∶000 dilution. YF 17D neutralizing mouse monoclonal antibody 8A3 [24] ascitic fluid was kindly provided by Jack Schlesinger (University of Rochester). A dilution of 1∶100 was found to efficiently neutralize up to 10^5^ pfu of YFV (data not shown). HRP-conjugated secondary anti-mouse (Jackson ImmunoResearch, 115-035-146) and anti-rabbit (Pierce, 31462) IgG antibodies were utilized at 1∶20,000 dilution. Normal rabbit IgG used in immunoprecipitations was from Santa Cruz Biotechnology, Inc.

### Viruses, virus titration and electroporation

YFV stocks were generated by electroporation of BHK-J cells as previously described [25] with in vitro transcribed YFV RNA. Plasmids pACNR-FLYF17Dx [12], pYF17D(5′C25Venus2AUbi), and pACNR-FL-YF-Asibi were used for generation of YF 17D, YFV-Venus or YF Asibi, respectively. All work with YF Asibi was conducted under Biosafety level 3 containment conditions. Virus stocks and samples were titered by infection of BHK-J cells with 10-fold serial dilutions in MEM with 2% FCS. Two hundred µl of diluted virus was added to each 35 mm well and after 1 h of infection the well was overlaid with 0.6% agarose in MEM supplemented with 2% FBS. Plaques were enumerated by crystal violet staining after 72 h. For YFV infections, multiplicity of infection (moi) was based on titers obtained on BHK-J cells.

HCVcc (Jc1FLAG2(p7-nsGluc2A)) a cell culture-derived HCV expressing *Gaussia* luciferase was prepared by electroporation of Huh7.5 cells as described previously [26].

Stocks of Kunjin (derived from infectious clone FLSDX 250pro) were propagated on Vero cells as described [27], [28]. Langat (TP21 strain) was propagated on Vero cells as described [29]. Titrations of stocks and samples were performed by focus forming assay, as previously described [30], [31]. Briefly, Vero cells were infected with 10-fold serial dilutions and after the 1 h adsorption the wells were overlaid with 0.8% methylcellulose in DMEM containing 2% FBS. After 4 d the monolayers were fixed with 100% methanol and plaques were visualized by incubation with polyclonal mouse antibody cross-reactive to Langat (hyperimmune mouse ascites fluid, clone Russian Spring Summer Encephalitis VR79; ATCC) or polyclonal mouse anti-West Nile virus E protein (obtained from Dr. Robert Tesh, World Reference Center for Emerging Viruses and Arboviruses) followed by secondary goat anti-mouse peroxidase-labelled polymer (DAKO Envision Systems) and application of peroxidase substrate containing 0.4 mg/ml 3,3′ diaminobenzidine and 0.0135% hydrogen peroxide in PBS.

To bypass entry steps, SW13 cells were electroporated essentially as described [32] with in vitro transcribed RNA generated from plasmid pACNR-FLYF17Da, YF-R.luc2A-RP, or pYF-R.luc2A-RP-ΔDD.

### Preparation of lentiviral stocks and transduction of cells

Stocks containing VSV-G pseudotyped lentiviral particles were generated essentially as described [7] by cotransfection using Fugene 6 (Roche) of 293T cells with plasmids encoding VSV-G, HIV gag-pol and the lentiviral provirus plasmid at a ratio of 1∶4∶4 µg. Medium overlaying the cells was harvested at 48–72 h after transfection, filtered through a 0.45 µM filter, aliquoted and stored at −80°C. Transductions were performed by incubating cells with the pseudoparticles in the presence of 8 µg/ml polybrene. The tissue culture 50% infectious dose (TCID_50_) was determined essentially as described [33] by titration on the TZM HeLa cell derivative [34], which expresses β-galactosidase under the control of the HIV LTR. Comparison of the TCID_50_ of a VSV-G pseudotyped V1-GFP stock, as determined on TZM cells, to the number of GFP-positive cells obtained after transduction of the target cell line with the same V1-GFP stock allowed for calculation of the appropriate TCID_50_ to utilize to achieve the desired transduction efficiency. For protein expression, transduction efficiency was typically in the range of 70–95%.

### Hamster cDNA library construction

A cDNA library was generated as described [7] from mRNA isolated from a BHK-21 derivative cell line, designated BHK/NZAP-Zeo [21], expressing the amino terminal fragment of the rat zinc-finger antiviral protein, after treatment for 6 h with 100 U/ml Universal type I IFN (PBL Biomedical Laboratories). Briefly, total RNA was harvested with Trizol (Invitrogen) and mRNA isolated by oligo dT selection (Oligotex mRNA Maxi Kit, Qiagen) according to the manufacturer's recommendations. The cDNA synthesis was carried using the method of the SMART cDNA Library Construction Kit (Clontech) with the outlined modifications [7] which utilized Superscript III (Invitrogen) for first strand synthesis and TaqPlus Long PCR System (Stratagene) for second strand synthesis and amplification, SfiI digestion and size fractionation with cDNA Size Fractionation Columns (Invitrogen). After ligation to the minimal HIV provirus V1 vector that had been SfiI-digested, and electroporation of DH10B cells (Invitrogen), the library was divided into two sub-libraries (L1 and L2), each with >3,000,000 clones, for amplification. Plasmid DNA was isolated from the amplified libraries using a Qiagen MaxiPrep Kit, and VSV-G pseudotyped particles expressing the library cDNAs were generated as described above. Library L1 contained insert sizes ranging from ∼400 to ∼2,400 nt and was utilized for these studies.

### cDNA library screen with cyclic packaging rescue

**Figure 1:**
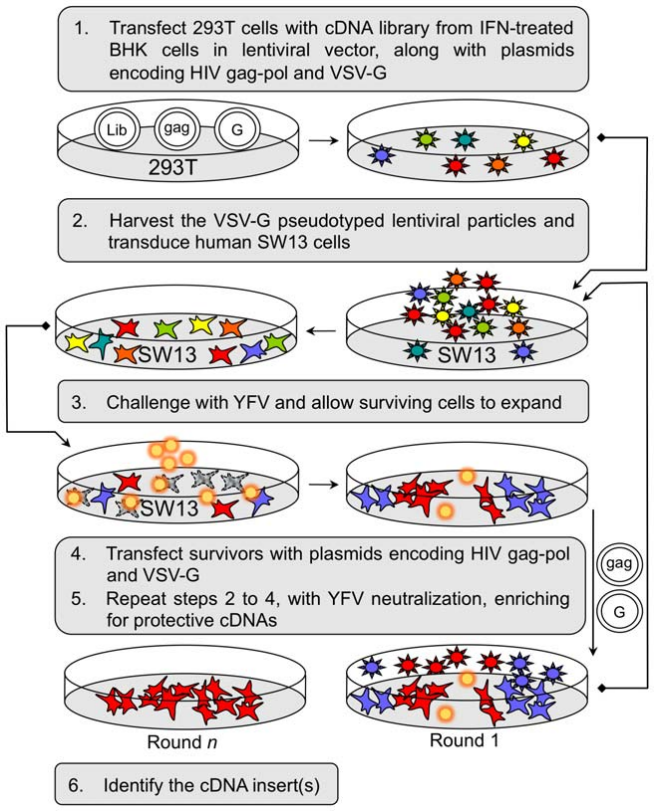
Selection and identification of host factors conferring protection against YFV-induced cell death. A schematic of the iterative selection process is shown.

**Figure 2:**
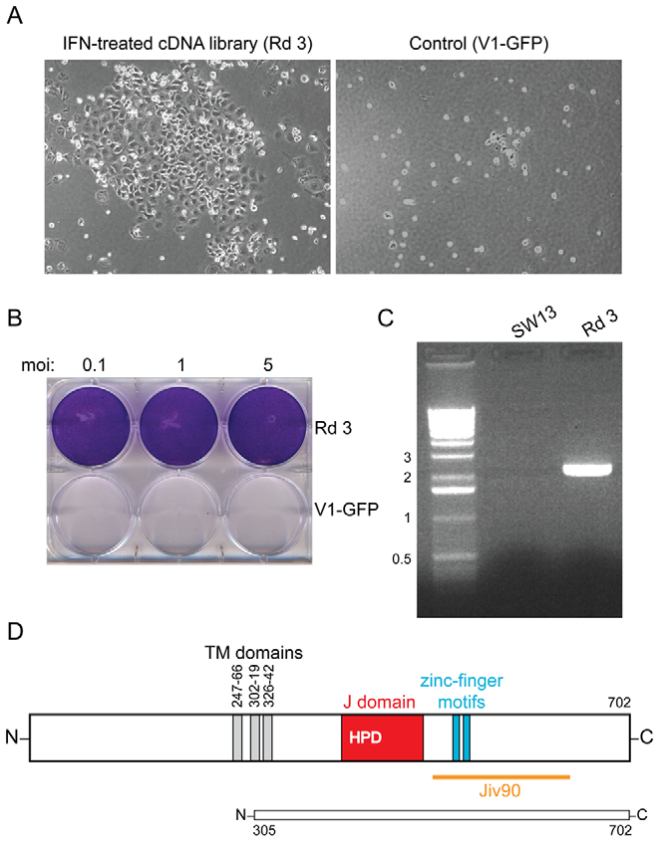
DNAJC14 confers resistance to YFV-induced cell death. (A) Photographs 7 d after YFV challenge (moi = 1) of SW13 cells transduced with Round 3 of the selected lentiviral cDNA constructs compared to cells transduced with V1-GFP vector control. (B) The cells transduced with the Round 3 lentivirus pool and surviving YFV infection (Rd 3) were expanded and reinfected with YFV at the indicated moi. Crystal violet staining was performed 3 d later. Cells transduced with vector alone serve as a control (V1-GFP). (C) DNA was isolated from naïve SW13 or Round 3 (Rd 3) cells, and the lentiviral insert amplified by PCR. The major band was identified as encoding a truncated hamster DNAJC14. Sizes of the DNA markers (kb) are indicated to the left. (D) A schematic of human DNAJC14 is shown, with the putative transmembrane (TM) domains (gray), J domain (red) with conserved HPD sequence, zinc finger motifs (blue) and Jiv90 domain (orange) indicated. A schematic of the isolated hamster clone, showing homology to amino acids 305 to 702 of human DNAJC14, is shown below.

The L1 cDNA library was screened for cDNAs able to confer resistance to YFV 17D-mediated cell death using a previously described iterative approach [7] and as outlined in Figure 1. SW13 cells (18 million) were transduced with the L1 library of lentiviral particles (0.45 TCID_50_/cell) and two days later were challenged with YFV 17D (moi = 5). After maintenance in MEM with 2% FBS for 7 d, the surviving Round 1 (Rd 1) cells were pooled and expanded in growth medium. The cDNA clones present in the surviving Rd 1 cells were rescued by transfection of cells in two 10 cm dishes with 15 µg VSV-G- and 5 µg HIV gag-pol-encoding plasmids diluted in OptiMem containing 40 µl Lipofectamine 2000 (Invitrogen)-according to the manufacturer's recommendations. The medium overlying the cells was collected 2 d later, pooled, filtered through a 0.45 µM filter, aliquoted and stored at −80°C. For subsequent steps, the rescued lentiviral stocks were treated for 2 h at room temperature with a 1∶100 dilution of mouse monoclonal 8A3 YFV neutralizing antibody prior to transduction of naïve SW13 cells in order to prevent cell death mediated by residual YFV in the lentiviral stock. Each rescued stock was utilized undiluted for subsequent transductions. Two additional rounds of transduction and challenge were performed to generate Rd 2 and Rd 3 cells and rescued lentiviral stocks. Rd 2 was performed on ∼4×10^6^ SW13 cells, (0.0003 R1 TC1D_50_/cell), and a YFV 17D challenge (moi = 5) 2 d later. Rescue was performed on the surviving Rd 2 cells in three 35 mm dishes, using Lipofectamine 2000-mediated transfection as described above (13 µl reagent, with 3 µg VSV-G- and 1 µg HIV gag-pol-encoding plasmids per dish). Rd 3 was performed on ∼2×10^6^ SW13 cells (0.07 R2 TC1D_50_/cell) and a YFV 17D challenge (moi = 1) 2 days later. A large number of cells survived the challenge compared to cells transduced with V1-GFP and challenged in parallel (see Figure 2A) and these Rd 3 cells were expanded for further testing. Rescue was performed as in Rd 2. A repeat experiment using the same Rd 2 rescued lentiviral stock gave similar results and an additional selection round using particles rescued from the Rd 3 cells also yielded many surviving Rd 4 cells (not shown).

### Identification of the cDNA conferring resistance to YFV

The predominant cDNA present in the Rd 3 cells was isolated by PCR. Rd 3 cellular DNA was isolated using the DNeasy Blood and Tissue Kit (Qiagen) and amplified using the Expand High Fidelity PCR System (Roche) and primers flanking the cDNA insert in the V1 vector (5′-GATTGTAACGAGGATTGTGGAACTTCTGGG-3′ and 5′-GATCCACAGATCAAGGATATCTTGTCTTCTTTGGG-3′). The PCR product was digested with SfiI, recloned into the V1 vector and sequenced using the above primers, as well as with primers designed to bind within the DNAJC14 sequence (5-TTGAAGCCACAGCATCC-3′ and AAGTCTACAGCTGCTCGAG-3′). Blast analysis demonstrated high homology with murine and human DNAJC14 with the cDNA insert predicted to express an amino-terminally truncated form of the protein. The nucleotide sequence of the truncated hamster DNAJC14 cDNA was submitted to GenBank (BankIt1399336 DNAJC14 HQ415606).

### Immunoprecipitation

To demonstrate DNAJC14 self interaction, HEK293T cells were seeded 16 h before transfection onto 60 or 100 mm tissue culture dishes at a density of 1.6×10^6^ or 4×10^6^ cells, respectively. The cells were co-transfected using Fugene 6 (Roche) with 2 (60 mm dish) or 4 (100 mm dish) µg each of pTrip-EGFP-hDNAJC14-NT5 and pV1-hDNAJC14-FL or mutants. Forty-eight hours post transfection, cells were scraped into ice-cold PBS and solublized with lysis buffer (10 mM HEPES, pH 7.5, 150 mM KCl, 3 mM MgCl_2_, 0.5% NP-40, 1×Proteinase inhibitor cocktail (Roche)), using 300 or 600 µl for 60 or 100 mm dishes, respectively. After disruption by passing through a 27G needle 5 times and clarification by centrifugation at 15,000×g for 10 min at 4°C, 300 µl of the soluble fraction was incubated overnight at 4°C with anti-myc, anti-GFP or control antibody. Pre-equilibrated protein A/G-agarose beads (Santa Cruz) were then added, and after 2 h of incubation, were collected by centrifugation and then washed four times with 600 µl washing buffer (10 mM HEPES, pH 7.5, 150 mM KCl, 3 mM MgCl_2_, 0.05% NP-40). The bound proteins were eluted by boiling in sodium dodecyl sulfate (SDS) sample buffer and were subjected to Western analysis.

To demonstrate the NS3-DNAJC14 interaction, SW13 cells were transduced with lentivirus expressing the myc tagged CT1 hDNAJC14 mutant and 2 d later were infected with YFV (moi = 1). After 2 d the cells were harvested and immunoprecipitation performed as described above except that the lysis and wash buffer contained 1% NP-40.

### Western blot analysis

Cells were directly lysed with 2×SDS loading buffer (100 mM Tris-Cl pH 6.8, 20% Glycerol, 4% SDS, 3% β-mercaptoethanol, 0.02% bromophenol blue) and boiled for 5 min. Proteins were separated by SDS-polyacrylamide gel electrophoresis (PAGE) and transferred to a Hybond ECL Nitrocellulose Membrane (GE Healthcare Life Sciences). The membrane was incubated in blocking buffer (PBS, 0.05% Tween 20, 5% dried milk) for 2 h, and then incubated with primary antibody diluted in blocking buffer at 4°C overnight. The membrane was washed 3 times in PBS supplemented with 0.05% Tween 20 and incubated for 2 h at room temperature with HRP-conjugated secondary antibody. After 3 washes, the membrane was visualized by ECL Supersignal West Pico (or Femto) Chemiluminescent substrate (Thermo scientific).

### Immunofluorescence and confocal microscopy

Cells were fixed with 4% formaldehyde in PBS and permeablized with 0.2% Triton X-100 in PBS for 5 min at room temperature. After being washed with PBS, samples were then blocked and incubated overnight with primary antibody in 3% BSA in PBS at 4°C After three washes with PBS, samples were incubated at 37°C for 1 h with Alex488- or Alex594-conjugated secondary antibody. Coverslips were finally mounted with Mowiol Mounting Media [0.1 M Tris-HCl, pH 8.5, 25% glycerol, 10% Mowiol 4–88 (Calbiochem 475904)] and observed by Leica LSM510 confocal laser with a 100×NA 1.3 oil immersion objective. Images were captured using the LSM software and processed using ImageJ.

### Flow cytometry

Cells were harvested by trypsinization, resuspended in PBS with 1% BSA, and then fixed in 2% formaldehyde in PBS. Samples were analyzed for expression of RFP and Venus using a BD LSR II flow cytometer, analyzing 10,000 events per sample. Data were processed using the FlowJo software.

### Luciferase assays

For the luciferase activity assay, transduced and infected Huh7.5 cells were washed twice with PBS and lysed with 1× Passive Lysis Buffer (Promega) according to the manufacture's recommendations. Luciferase activity was measured using the Renilla Luciferase Assay system (Promega) using a Lumat LB9507 Luminometer (Berthold).

### RNAi-mediated silencing and quantitative RT-PCR

Triplicate wells of SW13 cells transduced with V1-GFP control or DNAJC14-expressing lentiviruses were seeded in 24 well plates at 1×10^5^ cells per well in the presence of 60 nM Stealth RNAi siRNA Negative Control Med GC (12935-300) or DNAJC14-targeting Stealth siRNA (CCGAGGAACUAUGUCAACUUGGACA) and Lipofectamine RNAiMAX (Invitrogen) according to the manufacturer's reverse transfection protocol. The siRNA transfection was repeated 2 d later, using forward transfection with 60 nM siRNA. After an additional 2 d incubation, the cells were infected with YFV (moi = 5) and 24 h later the medium was collected for virus titration. For each condition, cells from one of the triplicate wells were harvested for Western blot analysis, while the remaining 2 wells were pooled for RNA harvest. RNA was purified using the RNeasy minikit (Qiagen) and each sample was reverse transcribed in triplicate using random primers and the Superscript III first strand synthesis kit (Invitrogen). Quantitative PCR was performed using the QuantiTect SYBR Green PCR Kit (Qiagen) and a LightCycler 480 (Roche) for detection as previously described [35]. Qiagen QuantiTect primers (QT00197043) were used for DNAJC14 amplification; levels were normalized to those of GAPDH, using a GAPDH primer set (sense: CCCACTCCTCCACCTTTGAC, antisense: CATACCAGGAAATGAGCTTGACAA) as described [36].

## Results

### Truncated hamster DNAJC14 inhibits YFV-mediated cell death

To identify cellular factors with antiviral activity against flaviviruses, we initiated a screen for host proteins that could inhibit cell death caused by YFV infection. We reasoned that a cDNA expression library generated from cells treated with interferon (IFN)-α to increase expression of antiviral factors would represent both IFN induced and constitutively expressed factors, some of which might have a protective effect against YFV. For this study, we utilized a cDNA library that we had generated (for other unrelated studies) from a BHK-21 cell derivative previously shown to develop dramatic resistance to Sindbis virus infection upon treatment with IFN [21]. Although we had some concerns regarding possible species incompatibilities for the function of hamster proteins in cells of other species, we thought it likely that factors influencing YFV, which has conserved replication strategies in both vertebrate and invertebrate cells, would function in a broad range of cells. We transduced YFV-susceptible human SW13 cells with the expression library, challenged these cells with YFV (vaccine strain 17D), and identified the cDNA(s) expressed in cells that survived the infection. During initial screens, we encountered several challenges, including difficulty cloning out rare surviving cells, and the presence of multiple library integrants. In order to overcome these obstacles, we expressed the cDNA library using a lentiviral vector (V1) in cells that are amenable to repackaging, as has been previously described [7]. Transfection of cells surviving the YFV challenge with helper plasmids expressing HIV gag-pol and an envelope glycoprotein (VSV-G) allows packaging of the lentiviral genomes, generating a lentivirus stock enriched for genes that confer a selective advantage (Figure 1). This approach obviates the need to clone individual cells, and allows iterative cycles of library transduction, YFV challenge, and rescue of sequences conferring survival. A YFV neutralization step of the selected, rescued lentiviral particles was used to prevent cell death mediated by residual virus during transduction of the naïve SW13 cells. After multiple rounds, the pool of cDNA-expressing lentiviruses will have markedly reduced complexity and active cDNA clones will be highly enriched.

After two rounds of selection, transduction of SW13 cells with the enriched library of lentiviral constructs resulted in extensive resistance to YFV-induced cell killing (Figure 2A). These “Round 3” (Rd 3) cells were expanded, retested for their susceptibility to YFV-induced cytopathicity and found to be resistant at several multiplicities of infection (moi, Figure 2B). DNA was harvested from the Rd 3 cells, and the cDNA inserts were amplified using primers specific for the V1 vector (Figure 2C). The single major PCR product (∼2.5 kb) was cloned, sequenced and found by BLAST analysis to show high homology to a murine (as well as human) Hsp40 family member, DNAJC14. The cDNA was predicted to express an N-terminally truncated version of the protein, which, based on the human sequence, lacked the first 304 amino acids of the 702 amino acid protein. DNAJC14 and the truncated hamster clone are shown schematically in Figure 2D.

To test whether the DNAJC14 sequence obtained by PCR could confer resistance to YFV-mediated cell death in naïve cells, the PCR product was cloned back into the V1 lentiviral vector. Five individual clones (designated 1-1, 1-2, 1-3, 1-4, and 1-5) were packaged and the VSV-G pseudotyped lentiviral particles were used to transduce naïve SW13 cells, which were challenged with YFV. Clone 1-1 was unable to confer resistance to YFV-mediated cell death, while each of the remaining four clones resulted in protection (data not shown). Sequencing of clones 1-1 and 1-2 indicated that both encoded a 398 amino acid protein (equivalent to human DNAJC14 aa 305–702) but clone 1-1 encoded a leucine to proline mutation at position 466, likely introduced during PCR amplification. This leucine is within the highly conserved J domain and is conserved amongst human, chimp, dog, cow, mouse and rat sequences. We conclude from these studies that expression of amino acids 305–702 of hamster DNAJC14 is able to confer resistance to YFV-mediated cell death.

Because our screen used cDNA from IFN-α-treated cells, we tested whether interferon treatment of SW13 cells results in upregulation of DNAJC14 mRNA levels. After treatment for 8 h with IFN-α, DNAJC14 RNA levels were quantified by real time RT-PCR. No significant differences in DNAJC14 RNA levels were found in cells treated with doses of IFN-α ranging from 0 to 1000 IU/ml (data not shown). Detection of eIF2α phosphorylation by Western blot demonstrated that IFN was active in these cells (data not shown). Although we cannot exclude that the gene may be upregulated in response to IFN-α in some cell types, *DNAJC14* appears to be constitutively expressed in SW13 cells.

### DNAJC14 mediates cell protection by inhibition of viral replication

**Figure 3:**
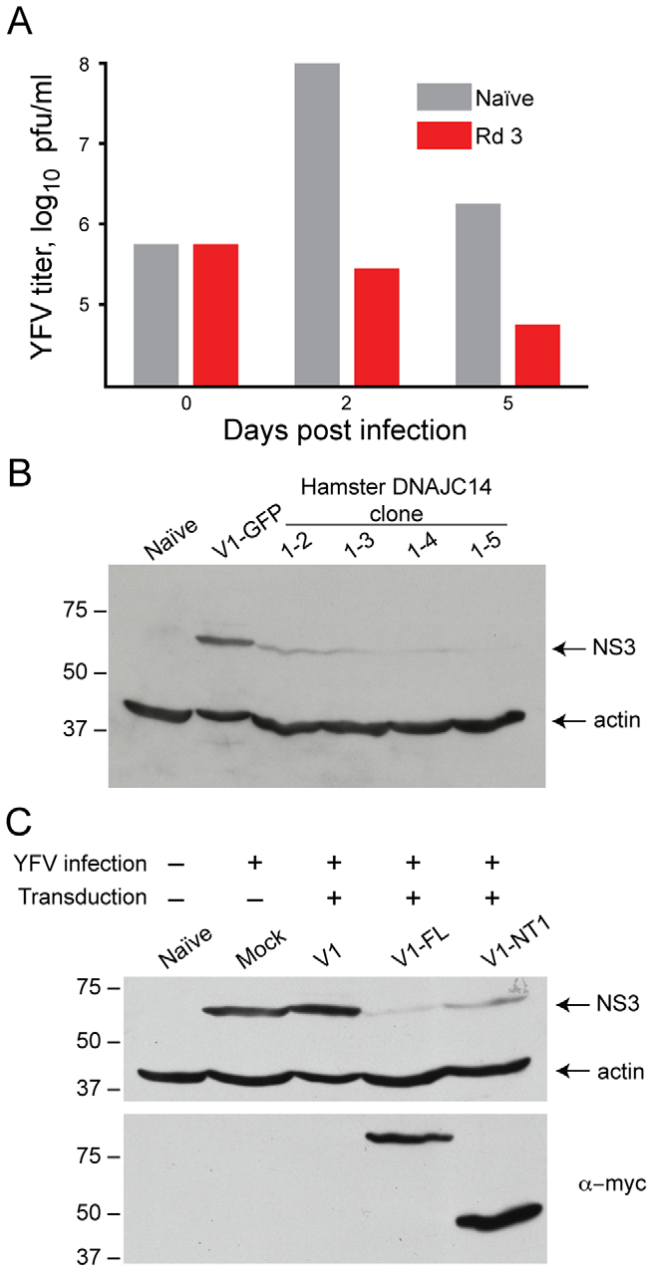
DNAJC14 inhibits YFV replication. (A). YFV replication is inhibited in Round 3 (Rd 3) cells. Rd 3 or naïve cells were infected with YFV (moi = 1) and at the indicated days after infection the amount of virus released into the medium was determined by plaque assay. A single well was used for each cell type, with replacement of the medium at each timepoint. (B). Hamster DNAJC14 inhibits YFV protein expression. SW13 cells were transduced with control lentivirus (V1-GFP) or lentivirus containing the truncated hamster DNAJC14 insert (clone 1-2, 1-3, 1-4 and 1-5) and were infected 2 d later with YFV (moi = 0.5). Cells were analyzed by Western blot for NS3 expression 3 d after infection. Naïve cells serve as a negative control and actin serves as a loading control. (C). Full-length and truncated human DNAJC14 inhibit YFV replication. SW13 cells were mock transduced (Mock) or transduced with the indicated lentiviral vectors and 2 d later were infected with YFV (moi = 0.5) as indicated. Cells were harvested and subjected to Western blot analysis 3 d after infection using antibodies as indicated to the right. V1-FL expresses myc epitope tagged full-length human DNAJC14 while V1-NT1 expresses myc tagged truncated human DNAJC14 (aa 305–702). Naïve cells were left untransduced and uninfected and serve as a negative control. For B and C migration of size standards (in kDa) is indicated.

Our screen utilized protection from viral-mediated cell death as an endpoint for the isolation of host proteins with activity against YFV. Survival could be due to inhibition of virus replication or prevention of activation and/or blocking of cell death pathways. To test whether expression of DNAJC14 resulted in inhibition of viral growth, we infected the Rd 3 cells with YFV and quantified virus production (Figure 3A). Compared to naïve cells, YFV propagation was markedly reduced in DNAJC14 expressing cells, with a greater than 2 log reduction in infectious titers at 48 h, and virus production continuing to decrease over time. In contrast, robust replication occurred at 48 h in the naïve cells, with infectious titers decreasing at 5 d, at which time the cells displayed massive cytopathic effect. To determine whether the decreased infectious titers were a result of decreased intracellular viral replication, we performed Western blot analysis (Figure 3B) on YFV-inoculated cells transduced with V1 vector containing the clone 1-2, 1-3, 1-4 and 1-5 inserts. We found reduced levels of the viral NS3 protein in the cells expressing hamster DNAJC14 compared to control cells transduced to express GFP. These results demonstrate that truncated hamster DNAJC14 blocks YFV infection and/or replication, which results in prolonged cell survival.

The truncated hamster DNAJC14 isolated in our screen is highly homologous to murine and human DNAJC14 proteins (89% identical and 93% similar to the corresponding region of the proteins). We therefore determined if expression of the 702 amino acid human DNAJC14 protein, also designated dopamine receptor interacting protein (DRIP78), could also confer protection against YFV-mediated cell death. We generated V1 lentiviral expression constructs to for both the full-length hDNAJC14 (hDNAJC14-FL) as well as an amino terminal truncation mutant (designated NT1) expressing amino acids 305–702 of human DNAJC14 (hDNAJC14-NT1), which corresponds to the hamster protein identified in our screen. A C-terminal myc epitope tag was engineered in the constructs to allow detection of the proteins. After packaging, the lentiviral pseudoparticles were used to transduce SW13 cells, which were then challenged with YFV (Figure 3C). Both the full-length and truncated versions of DRIP78 inhibited intracellular YFV NS3 accumulation. These studies demonstrate that the human DNAJC14 homolog is able to inhibit YFV infection and/or replication, as well as show that the addition of a C-terminal epitope tag does not interfere with the inhibitory activity.

### DNAJC14 is a broad-spectrum *Flaviviridae* family replication modulator

**Figure 4:**
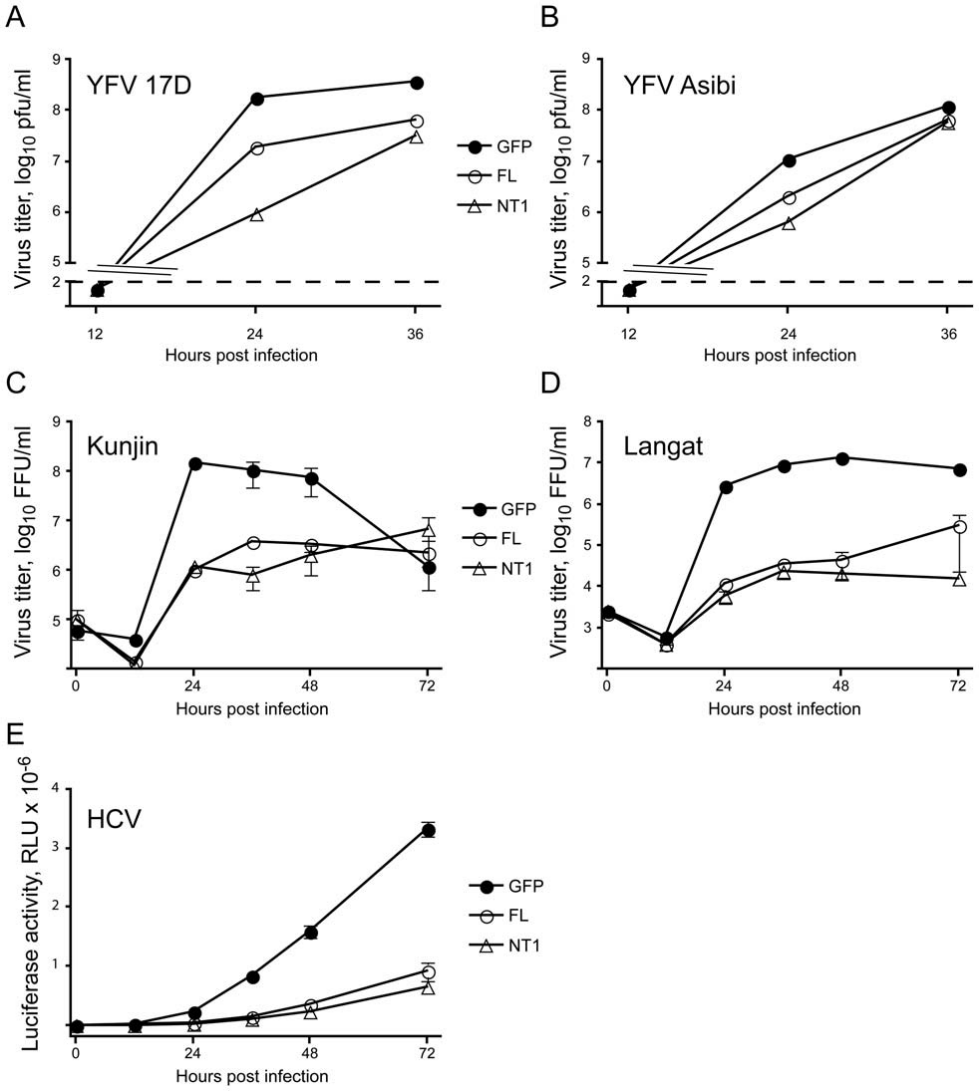
DNAJC14 inhibits multiple members of the *Flaviviridae* family. SW13 (A, B) or Huh7.5 (C–E) cells were seeded in equal numbers into plates and transduced with V1 vector expressing GFP (GFP, filled circles), V1 vector expressing full-length human DNAJC14 (FL, open circles) or V1 vector expressing truncated (aa 305–702) human DNAJC14 (NT1, triangles). After 2 days, the cells were challenged (moi = 5) with YFV 17D (A), YFV Asibi (B), Kunjin virus (C), Langat virus (D) or were challenged (moi = 0.1) with HCV Jc1FLAG2/p7-nsGluc2A. The medium (A–D) or cells (E) were harvested at the indicated times for quantification of virus replication. For A and B, a single separate well was utilized for each time point, and virion production was enumerated by plaque assay. In both cases, there were fewer than 100 plaque-forming units at 12 h post infection. The dashed line indicates the sensitivity of the plaque assay. Pfu, plaque forming units. For C and D, duplicate wells were infected and the medium was harvested and replaced at each timepoint. Virion production since the prior time point was enumerated by focus forming assay as described in Materials and Methods. Data points represent the mean titer; error bars indicate the range. Similar results were obtained in an independent experiment for both Kunjin and Langat viruses. FFU, focus forming units. For E, cells were harvested at the indicated times after infection for measurement of luciferase activity as described in Materials and Methods. Data points represent mean values obtained from triplicate wells; error bars indicate the standard deviation. RLU, relative light units.

Bovine DNAJC14 (also known as J-domain protein interacting with viral protein, or Jiv) has previously been implicated in regulation of pestivirus (BVDV) replication. Intriguingly, cytopathic strains of the virus can contain insertions of DNAJC14 within their genome [37], [38], [39]. A portion of the Jiv protein (Jiv90) is required for the substrate interaction and activity of the viral autoprotease (NS2) and the subsequent establishment of replication complexes [40], [41], [42]. We therefore wondered how expression of DNAJC14 would affect other members of the *Flavivirus* genus, as well as the *Hepacivirus* genus member HCV. We first compared the ability of DNAJC14 to inhibit YFV 17D (vaccine strain) and the prototype virulent Asibi strain isolated from a young Ghanaian patient in 1927 [43]. Measuring of infectious virus production by transduced cells indicated that both the vaccine and Asibi YFV strains were susceptible to inhibition by the truncated or full-length hDNAJC14 (Figure 4A and B). Kunjin, a more distantly related mosquito-borne *Flavivirus* genus member (from the Japanese encephalitis serocomplex group) was also found to be susceptible to hDNAJC14-mediated inhibition (Figure 4C). A representative of the tick-borne encephalitis group, Langat virus, was similarly susceptible to hDNAJC14's inhibitory effects (Figure 4D). It therefore seems likely that DNAJC14 broadly affects members of the *Flavivirus* genus. To establish whether hDNAJC14 also has effects on the *Hepacivirus* genus, we used HCV Jc1FLAG2(p7-nsGluc2A), a cell-culture infectious virus (HCVcc) expressing a luciferase reporter [26]. Again, both the full-length DNAJC14 and NT1 truncation mutant, corresponding to our isolated hamster clone, inhibited viral propagation. Taken all together, these results suggest that DNAJC14 modulates the replication of many or all members of the *Flaviviridae* family.

### DNAJC14 inhibits YFV infection at a post entry step

**Figure 5:**
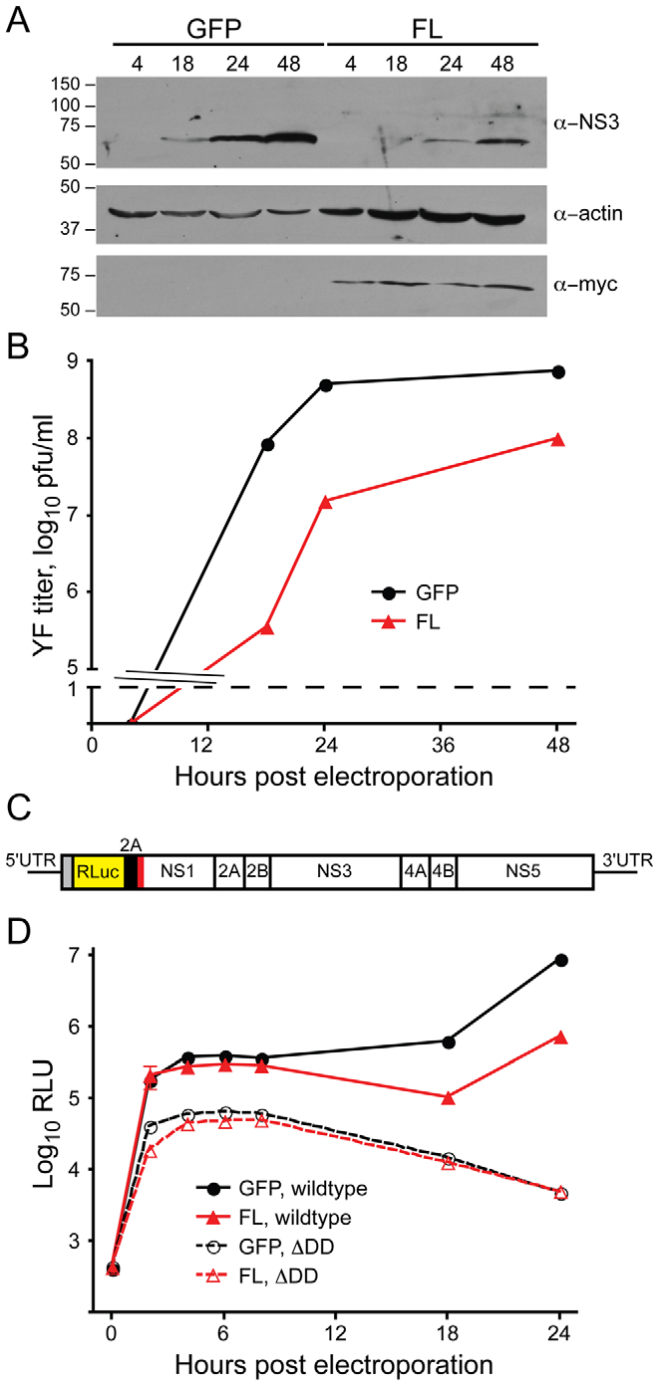
DNAJC14 inhibits a post entry step. (A) and (B) V1-GFP- (GFP) or V1-hDNAJC14-FL (FL) transduced SW13 cells were electroporated 2 d later with in vitro transcribed YF-17D RNA to bypass the entry step. Cells and media were harvested at the indicated times after electroporation. (A) Western blot analysis was performed on equal volumes of the cell extracts using the antibodies indicated to the right; actin serves as a loading control and antibodies to myc detect the tagged DNAJC14 protein. Migration of size standards (in kDa) is indicated to the left. (B) Virus present in the medium from V1-GFP- (GFP, black circles) and V1-hDNAJC14-FL (FL, red triangles) transduced cells was enumerated by plaque assay. A single separate well was utilized for each time point. Pfu, plaque forming units. (C) Schematic of the YFV replicon construct, which expresses Renilla luciferase (RLuc) in place of the structural proteins. The locations of the YFV nonstructural proteins are indicated in the polyprotein, which is targeted to the ER by a signal sequence (red bar). UTR, untranslated region; 2A (black bar), the foot and mouth disease virus 2A autoproteolytic peptide. (D) V1-GFP- (GFP, circles) and V1-hDNAJC14-FL- (FL, triangles) transduced SW13 cells were electroporated with the wild type YF replicon RNA (filled symbols, solid lines) or with replication incompetent RNA containing a mutation in the RNA-dependent RNA polymerase (ΔDD, open symbols, dashed lines). At various times after electroporation, the cells were harvested and luciferase activity was determined. Data represents the mean luciferase value of triplicate samples; error bars indicate the standard deviation and are sometimes obscured by the symbol. RLU, relative light units. Similar results were obtained in an independent experiment utilizing Huh7.5 cells.

Since DNAJC14 regulates dopamine D1 receptor transport [44], it is possible that inhibition of YFV is the result of disrupting the transport of a cell surface receptor(s) utilized by the virus. We introduced the YFV genomic RNA into cells by electroporation in order to bypass entry and to determine if downstream steps are affected by DNAJC14. We found that DNAJC14 was still able to mediate inhibition of YFV protein expression when entry steps are bypassed; infectious virion production was also reduced (Figure 5A and B). The reduced protein levels and virion production might be due to decreased translation, RNA replication, assembly and/or egress. Using a YFV replicon (Figure 5C) expressing luciferase in place of the structural proteins [17], we tested whether DNAJC14 results in reduced expression of viral protein. The results (Figure 5D, wildtype replicon) demonstrate that YFV translation levels are reduced at later time points in the DNAJC14-expressing cells. Since the replicon does not express the structural proteins and is incapable of spread, the results suggest that DNAJC14-mediated inhibition can occur at a step after entry and prior to assembly, egress and spread. Interestingly, luciferase expression at early time points after electroporation was similar in the control (V1-GFP) and DNAJC14-expressing cells, suggesting that genome translation is not inhibited by DNAJC14. At later times (after 8 h) increased luciferase activity was detected in the control cells, suggesting that new RNA had been synthesized for translation. Use of a replicon containing a mutation abolishing RNA-dependent RNA polymerase activity (ΔDD) demonstrates that translation of the genomic RNA is similar in DNAJC14 overexpressing and control cells. Taken together the results indicate that a step after entry and translation and before assembly and egress is affected by DNAJC14.

### Mutagenesis of DNAJC14 implicates the J domain and C-terminus in YFV inhibition

**Figure 6:**
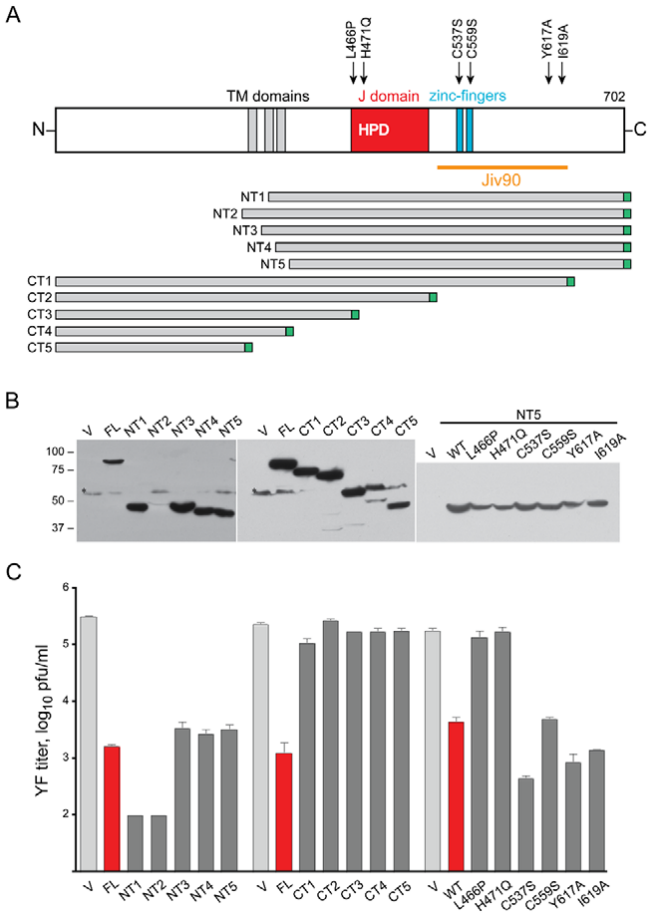
Mutagenesis of DNAJC14. (A) A schematic of DNAJC14 is shown with the truncation mutants indicated below. Arrows above the schematic indicate the location of point mutants engineered in the NT5 truncation mutant backbone. Each mutant contained a C-terminal myc epitope tag, indicated in green. (B and C) SW13 cells were transduced with lentivirus expressing the indicated mutants and 2 d later were challenged with YFV (moi = 5). Cells and media were harvested 1 d later. V, control V1 lentivirus; FL, lentivirus expressing full-length hDNAJC14; WT, the NT5 truncation mutant without any point mutations. (B) Western blot analysis was performed on equal volumes of the cell extracts using anti-myc antibody to detect the tagged DNAJC14 or mutant protein. Migration of size standards (in kDa) is indicated to the left. (C) YFV present in the medium was enumerated by plaque assay. Data represent mean values obtained from triplicate wells; error bars indicate the standard deviation. Pfu, plaque forming units.

To ascertain determinants of DNAJC14 inhibitory function, we generated deletion and point mutants and tested their ability to inhibit YFV infection. A schematic of the deletion mutants is shown in Figure 6A. Expression levels, as determined by Western blot detecting the C-terminal myc tag on each of the constructs, were variable (Figure 6B), although immunofluorescence analysis verified almost 100% percent transduction efficiency for each of the mutants (data not shown). DNAJC14 has been proposed to reside in the ER membrane with both its N and C termini located within the cytoplasm [44]. This predicted topology was based on the interaction of DNAJC14 with the C terminus of the dopamine D1 receptor, as well as on DNAJC14 hydrophobicity plots and the absence of a signal peptide. Topology prediction programs suggest three potential regions that may serve as transmembrane (TM) domains. The truncated hamster mutant identified in our screen (NT1) contains an amino terminal deletion and is predicted to have one TM domain. Of the N terminal deletion mutants, NT3, NT4 and NT5 (lacking one, two or all three potential TM domains) exhibited similar antiviral activity to the full-length protein, while NT1 (lacking two) and NT2 (containing all three TM domains) exhibited the most potent activity (Figure 6C). Thus while the most inhibitory mutants contained at least one putative TM domain, the presence of a TM domain is not strictly required for inhibition. The C terminal deletion series were uninformative with respect to the role of the TM domains, since deletion of the C terminal 77 amino acids of DNAJC14 (mutant CT1), and various further deletions (mutants CT2, CT3, CT4, and CT5, which lacks all 3 TM domains) all resulted in a protein devoid of antiviral activity (Figure 6C). This suggests the carboxyl terminal 77 amino acids of DNAJC14 are required for antiviral activity. Although mutants CT3, CT4, and CT5 all contained deletions of the J domain, they also were not informative as to the role of the J domain in antiviral activity, since they also lacked the important C terminal domain.

We utilized the NT5 mutant, which has robust expression and inhibitory activity equivalent to wildtype hDNAJC14, as the backbone to test several point mutations for their affect on YFV inhibition (Figure 6B, C). Our initial hamster clone 1-1 construct contained a presumed PCR-induced mutation at leucine 466 (to proline) within the J domain and was unable to confer resistance to YFV (not shown). We tested the L466P mutation in the context of hDNAJC14-NT5 and found that it abrogated the antiviral activity against YFV, suggesting a role for the J domain in the inhibitory process. Within the J domain, the conserved HPD motif is important for accelerating the ATPase activity of Hsp70 [45] and mutation of this motif (mutant H471Q) resulted in a noninhibitory protein. Studies on the interaction of rat DNAJC14 with the dopamine receptor [44] implicate the zinc fingers within the Jiv90 domain as important to the dopamine receptor-DNAJC14 interaction; mutation of cysteine 536 (537 in human DNAJC14, Figure 6), to serine abolished the DNAJC14-dopamine receptor interaction. We therefore generated mutations in two of the conserved Jiv90 cysteine residues, predicted to be involved in zinc coordination. Interestingly, mutants C537S and C559S could still inhibit YFV infection. We also mutated two residues (Y617A, I619A) that are required for maximal bovine Jiv90-mediated stimulation of BVDV NS2-3 cleavage [40]. Interestingly, these two mutants also displayed potent anti-YFV activity. Taken all together, the results suggest that the J domain and C-terminal domain are important for DNAJC14's inhibitory effects on YFV. Both the DNAJC14 determinants important for modulation of pestivirus and flavivirus replication, as well as the result of DNAJC14 overexpression differ for viruses in these two *Flaviviridae* genera.

### The DNAJC14 carboxyl terminus mediates self-interaction and is required for antiviral activity

**Figure 7:**
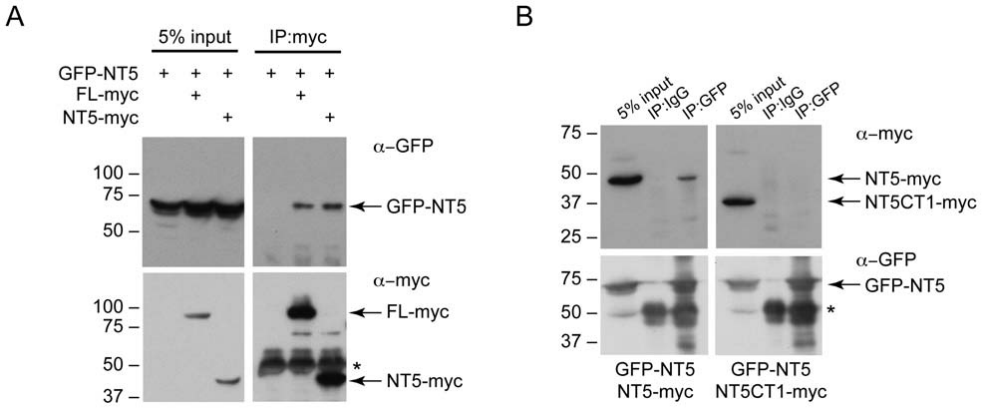
The C-terminus of DNAJC14 mediates self-interaction. (A) Self-interaction of DNAJC14. HEK293T cells were cotransfected with pTrip-EGFP-hDNAJC14-NT5 (GFP-NT5) and pV1-hDNAJC14-FL (FL-myc) or the NT5 mutant (NT5-myc) as indicated. Cells were harvested 2 d later and myc-tagged DNAJC14 was immunoprecipitated using anti-myc antibody. Western blots were performed using anti-GFP and anti-myc antibodies as indicated. (B). Self-interaction is mediated by the C-terminus. HEK293T cells were cotransfected as indicated with pTrip-EGFP-hDNAJC14-NT5 (GFP-NT5) and pV1-hDNAJC14-NT5 (NT5-myc, left panels) or the NT5CT1 mutant (NT5CT1-myc, right panels) lacking the C terminal 77 amino acids. Cells were harvested 2 d later and DNAJC14 was immunoprecipitated using anti-GFP or control IgG as indicated. Western blots were performed using anti-GFP and anti-myc antibodies as indicated. For both A and B, arrows indicate the DNAJC14 proteins; migration of size standards (in kDa) is indicated to the left. The asterisk indicates immunoglobulin heavy chain.

Hsp40 family members are categorized into three classes [45], [46], [47]. Type I proteins contain the J domain at the N terminus followed by a glycine/phenylalanine rich region, four zinc finger motifs and a peptide binding fragment, with a C-terminal dimerization domain. Type II proteins contain an amino terminal J domain, and C-terminal peptide binding fragment, but lack the zinc-finger motifs, while Type III proteins are variable, with the J domain localized anywhere in the protein. DNAJC14 would thus be categorized as a Type III Hsp40, although the presence of two zinc-finger motifs downstream of the J domain suggests some similarities to the Type I members. Structural and functional analyses of several Type I and Type II Hsp40 members [45], [46] demonstrate that the C-terminal domains mediate dimerization. We therefore investigated whether DNAJC14 was capable of self-interaction. Using the NT5 mutant, which contains the C-terminal region, we tested its ability to interact with itself and with full-length DNAJC14. SW13 cells were cotransfected with plasmids expressing GFP and myc tagged DNAJC14 proteins and immunoprecipitations were performed using anti-myc antibodies. GFP-tagged NT5 co-purified with myc-tagged DNAJC14 or NT5 during myc-mediated immunoprecipitation, demonstrating self-interaction (Figure 7A). The NT5 self-interaction was verified by the reciprocal immunoprecipitation using anti-GFP antibodies (Figure 7B, left panel). However, mutant NT5 lacking the C-terminal 77 amino acids (NT5CT1) failed to co-purify in the immunoprecipitation (Figure 7B, right panel). Thus, similar to the Type I Hsp40 members, DNAJC14 multimerizes, and the self-interaction is mediated by the C-terminal 77 amino acids. Since the CT1 mutant also fails to inhibit YFV, it is possible that multimerization (likely dimerization) is important for DNAJC14's antiviral activity.

### DNAJC14 does not inhibit YFV nor HCV polyprotein cleavage

**Figure 8:**
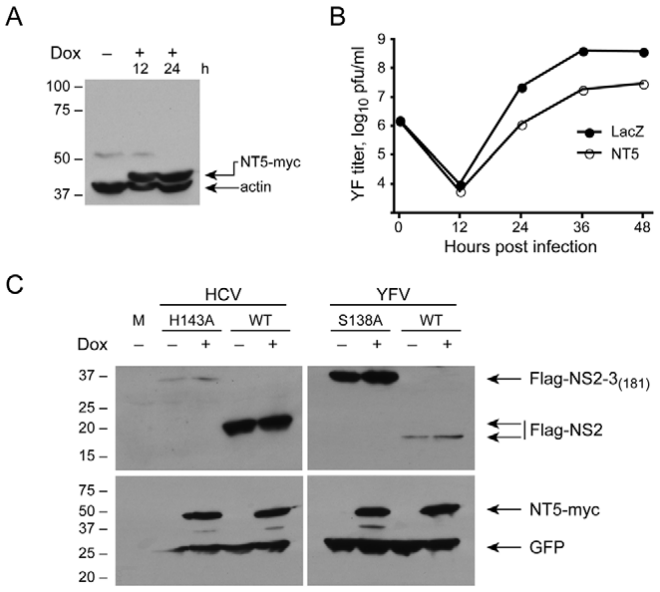
DNAJC14 does not inhibit NS2/3 cleavage of YFV and HCV. YFV replication is inhibited in cells inducibly expressing the NT5 mutant form of DNAJC14. (A) T-REx-293-NT5 cells were left uninduced or induced by treatment with doxycycline (Dox) for the indicated h and lysates were analyzed by Western blot using anti-myc and anti-actin (loading control) antibodies. Migration of size standards (in kDa) is indicated to the left. (B) T-REx-293-NT5 (NT5) or T-REx-293-LacZ (LacZ) cells were induced to express DNAJC14-NT5 or β-galactosidase, respectively, by 24 h treatment with doxycycline. The cells were then infected with YFV (moi = 5) and the medium was harvested and replaced at each timepoint. Virion production since the prior time point was enumerated by plaque assay. Pfu, plaque forming units. (C) YFV and HCV NS2/3 cleavage in T-REx-293-NT5 cells. T-REx-293-NT5 cells were left untreated or were induced to express DNAJC14-NT5 by 24 h treatment with doxycycline (Dox) as indicated. The cells were then cotransfected with pEGFP (loading control) and either pFlag-HCV-NS2/3(181) or pFlag-YFV-NS2/3(181). As controls, NS2-3 proteins containing active site mutations in the HCV NS2 (H143A) or YFV NS3 (S138A) proteases and incapable of cleavage activity were also expressed as indicated. Cells were harvested 1 day later and analyzed by Western blot using anti-Flag antibody (top panel) or anti-myc and anti-GFP antibodies (bottom panel). Arrows indicate the migration of the relevant proteins and migration of size standards (in kDa) is indicated on the left.

DNAJC14 is a required cofactor for the BVDV NS2 protease, which mediates autoproteolytic cleavage of NS2-3 as a necessary prerequisite for RNA replication [40]. Overexpression of DNAJC14 enhances cleavage at the 2/3 site, RNA replication and cytopathogenicity, but results in reduced infectious virion production due to a requirement for uncleaved NS2-3 for late life cycle events [48]. In contrast, in the case of YFV, DNAJC14 overexpression inhibits RNA replication (Figure 5D). Based on this apparent opposite effect, we wondered if DNAJC14 might inhibit (rather than enhance) YFV NS2B-3 cleavage and result in reduced levels of subsequent RNA replication. It is of interest that for YFV, cleavage at the NS2B/3 site is mediated by the viral NS3 protease, while for HCV the cleavage of the NS2/3 site is mediated by NS2. Since the effects of DNAJC14 on cleavage at the NS2/3 site of BVDV was successfully determined by coexpression of DNAJC14 and viral fragments capable of self-cleavage [39], we took a similar approach to test whether DNAJC14 inhibited YFV NS2B-3 cleavage. We first generated a doxycycline-inducible cell line expressing hDNAJC14 mutant NT5 with a C-terminal myc tag (Figure 8A). As expected, YFV replication was reduced in this cell line when treated with doxycycline to induce hDNAJC14-NT5 expression (Figure 8B). Using transfection, we expressed Flag-tagged self-cleavage competent YFV NS2B-3_181_ as well as a form incapable of cleavage due to a S138A active site mutation within NS3 [49] and monitored cleavage in doxycycline treated (expressing hDNAJC14-NT5) or non-induced control cells by Western blot. Similarly, we expressed Flag-tagged self-cleavage competent HCV NS2-3 protease, as well as a form incapable of autocleavage due to a H143A active site mutation within NS2 [11]. A plasmid expressing GFP was cotransfected to monitor transfection efficiency. As shown in Figure 8C, wildtype NS2B-3 or NS2-3 was efficiently processed resulting in similar levels of NS2 in the presence or absence of DNAJC14-NT5. The low levels of cleavage incompetent HCV NS2B-3 are likely due to the previously described rapid degradation of uncleaved NS2-3 [50]. Thus, contrary to our prediction, DNAJC14 does not grossly inhibit YFV, or HCV polyprotein cleavage. However, due to the sensitivity of this assay, subtle inhibition of processing efficacy would likely not be detected. Moreover, given the efficiency of cleavage in cells not induced to express DNAJC14, we cannot exclude an enhancement effect, similar to that seen with BVDV NS2-3 processing, of DNAJC14 on YFV NS2B-3 or HCV NS2-3 cleavage.

### DNAJC14 is recruited to YFV replication complexes

**Figure 9:**
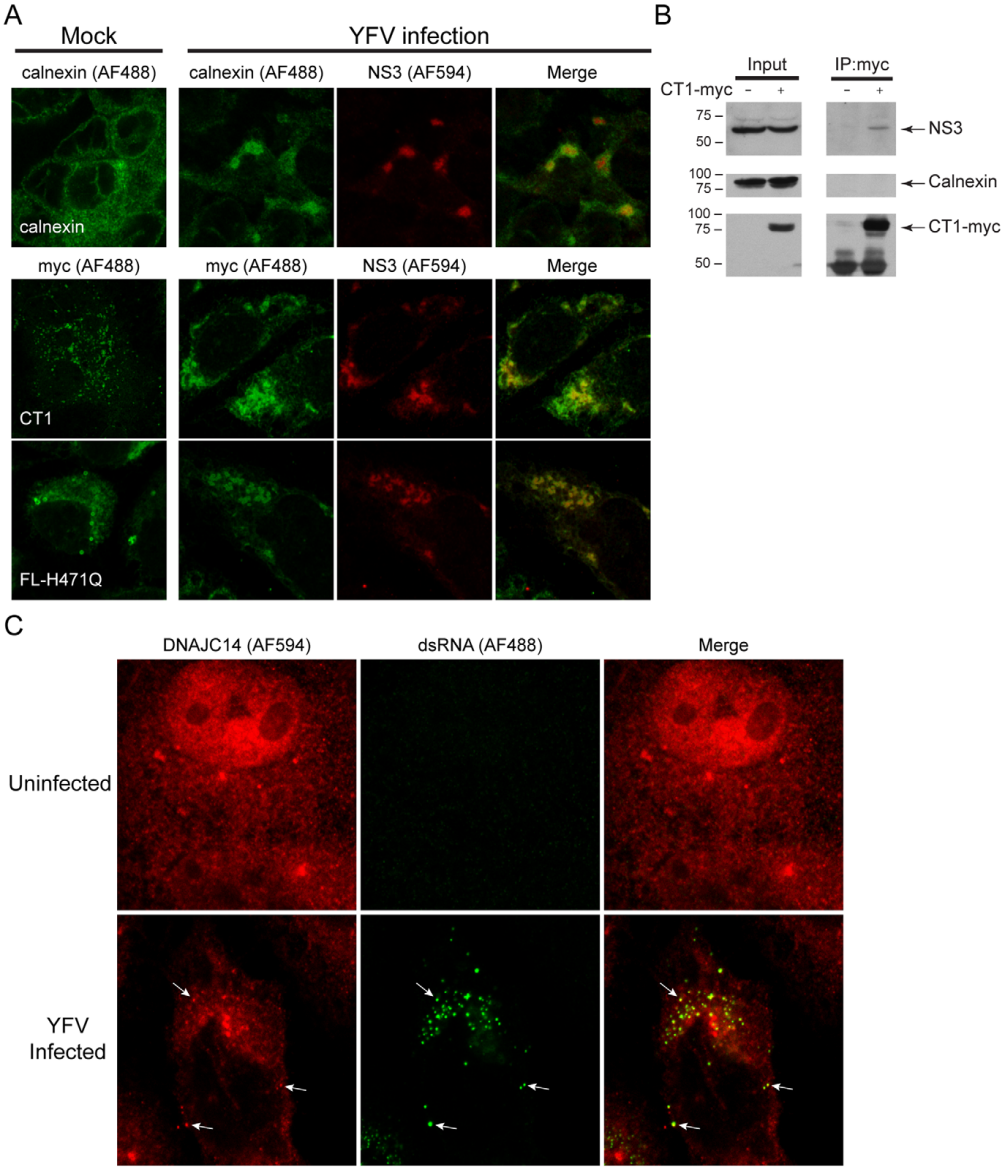
DNAJC14 is recruited to YFV replication complexes. (A) SW13 cells were left untransduced (top row) or were transduced (lower 2 rows) with the noninhibitory V1-hDNAJC14 mutants FL-H471Q or CT1 as indicated. Two d later the cells were mock treated (left panels) or were challenged with YFV (moi = 5, right 3 panels). After an additional 2 d, the cells were fixed and immunostained with rabbit anti-YFV NS3 polyclonal antibodies (NS3), and mouse anti-calnexin antibody (calnexin) or mouse anti-myc monoclonal antibody (myc) as indicated. AF488-conjugated anti-mouse IgG and AF594-conjugated anti-rabbit IgG antibodies were used as secondary antibodies. The cells were analyzed by confocal microscopy and representative images are shown. Calnexin or DNAJC14 mutants are shown in green, YFV NS3 is shown in red, and the merged images are shown on the right. (B) SW13 cells were left untransduced or were transduced with the V1-hDNAJC14-CT1 mutant (CT1-myc) as indicated and were infected 2 d later with YFV (moi = 1). After 2 d of infection, myc-tagged DNAJC14-CT1 was immunoprecipitated using anti-myc antibody. Western blots were performed using antibodies against NS3, calnexin and the myc epitope tag as indicated. (C) SW13 cells were left uninfected or were infected with YFV (moi = 1) as indicated. The cells were fixed 1 d later and analyzed by confocal microscopy for endogenous DNAJC14 (red) and double stranded RNA (dsRNA, green). The merged image is shown on the right. Arrows indicate several areas of colocalized DNAJC14 and dsRNA.

Since DNAJC14 does not inhibit YFV genome translation yet blocks RNA replication, we wondered if it might interfere with the formation of functional replication complexes, which assemble on ER-derived membranes. Studies to investigate whether hDNAJC14 colocalizes with replication complexes in YFV infected cells are complicated by the fact that DNAJC14 expression inhibits YFV replication. To determine whether hDNAJC14 colocalizes with YFV replication complexes, we made use of the non-inhibitory DNAJC14 mutants H471Q and CT1 and monitored their colocalization with YFV NS3. SW13 cells transduced with lentiviruses expressing hDNAJC14 mutants were infected with YFV and the localization of NS3 and hDNAJC14 was examined by confocal microscopy (Figure 9A). As a control, we examined the localization of calnexin and demonstrated that YFV infection results in a redistribution of this ER marker to colocalize with NS3 in infected cells (Figure 9A). Full-length DNAJC14 containing the J domain mutation H471Q (FL-H471Q) and mutant CT1 both colocalized with NS3 in infected cells. The results are consistent with the known ER reorganization that occurs during YFV replication complex formation and suggest that DNAJC14 proteins associated with the ER membrane redistribute to replication complexes during YFV infection. Expression levels of the CT1 mutant, which is recruited to sites containing NS3 (Figure 9A) without blocking replication (Figure 6C), are similar to expression levels of the inhibitory full-length protein (Figure 6B). This makes a nonspecific process, such as the induction of ER stress due to protein overexpression, unlikely for the inhibitory mechanism.

To assess further whether DNAJC14 associates with the viral replication complexes, we looked for a physical interaction using coimmunoprecipitation. Cells transduced (or not) to express the myc epitope tagged noninhibitory CT1 mutant were infected with YFV. CT1 and associated proteins were isolated from lysates using anti-myc antibody. As can be seen in Figure 9B, NS3 was coimmunoisolated with CT1, while the ER marker calnexin was not. Thus while both calnexin and CT1 colocalize with NS3 in immunofluorescence assays (Figure 9A), NS3, but not calnexin, was found to be in a physical complex with CT1. We utilized antibodies directed against dsRNA as another means to identify replication complexes and assess whether endogenous DNAJC14 is present (Figure 9C). Using anti-DNAJC14 antibody, we found that in uninfected cells, endogenous DNAJC14 in the cytosol predominantly displayed a diffuse pattern with occasional punctate staining. In infected cells the endogenous DNAJC14 demonstrated a more punctate staining pattern and the dsRNA was found colocalized with these punctate sites of staining. These findings demonstrate that both endogenous DNAJC14 and overexpressed non-inhibitory DNAJC14 mutants are recruited to YFV replication complexes, which suggests that endogenous DNAJC14 may facilitate replication complex formation.

### Endogenous DNAJC14 facilitates YFV

**Figure 10:**
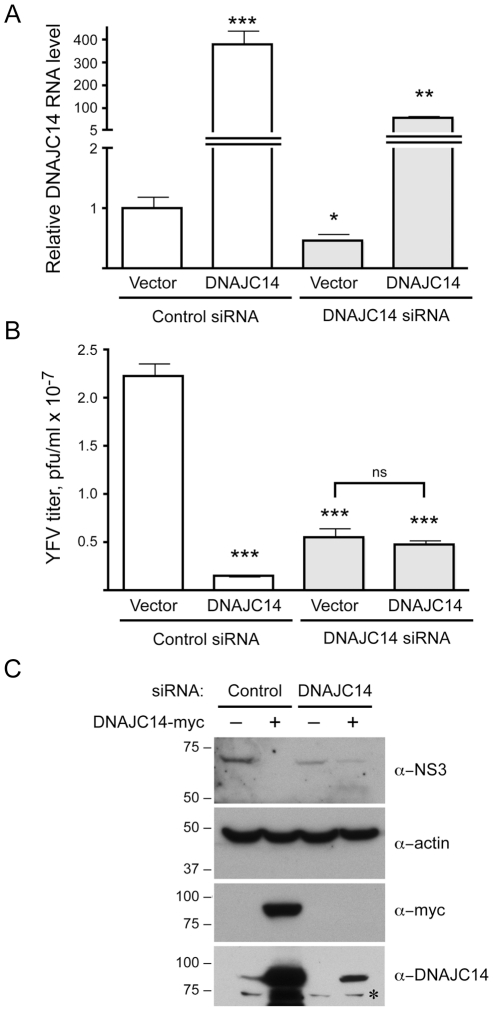
Modulation of DNAJC14 levels by siRNA alters YFV replication. SW13 cells transduced with V1-GFP (Vector) or V1-hDNAJC14-FL (DNAJC14) were treated with irrelevant control siRNA or siRNA targeting DNAJC14 as indicated and were infected with YFV (moi = 5). After 24 h the medium from triplicate samples was collected for virus titration, while 2 samples were pooled for RNA isolation and cells in the remaining sample were harvested for Western analysis. (A) DNAJC14 RNA levels were determined by quantitative RT-PCR. For each sample, DNAJC14 RNA levels were normalized to levels of GAPDH RNA and the ratio present in the vector control cells treated with the irrelevant control siRNA was set to 1. Bars represent mean relative levels obtained from triplicate RT reactions; error bars indicate the standard deviation. Asterisks indicate a significant difference from the cells transduced with control vector and receiving the control siRNA (students t test; *p<0.05, **p<0.01, ***p<0.001). (B) Virus present in the medium was titered by plaque assay. Bars represent mean titers from triplicate samples; error bars indicate the standard deviation. Pfu, plaque forming units. Asterisks indicate a significant difference in virus production compared to cells transduced with control vector and receiving the control siRNA (students t test; ***p<0.001). Virus titers obtained after silencing DNAJC14 in the vector control versus DNAJC14 transduced cells were not statistically different (ns). (C) Western blot analysis was performed on the silenced samples using the indicated antibodies. Whether the cells were transduced with V1-hDNAJC14-FL (DNAJC14-myc+) or control vector (−) is indicated. Migration of size markers (in kDa) is indicated to the left. The asterisk indicates a non-specific band.

To test whether DNAJC14 might be required for, or facilitate virus replication, we used siRNA-mediated silencing to reduce levels of endogenous DNAJC14 and tested the ability of YFV to replicate. To evaluate replication capacity across a range of DNAJC14 levels, we used cells transduced with vector as well as cells transduced with lentivirus expressing DNAJC14 and subjected them to silencing with a control irrelevant siRNA or siRNA targeting DNAJC14 mRNA within the protein coding region. It should be noted that the absolute level of DNAJC14 RNA in normal cells (vector-transduced cells treated with control siRNA) is low, with DNAJC14 RNA levels more than 1000 fold lower than GAPDH mRNA levels (data not shown). Reducing levels of DNAJC14 mRNA by ∼2 fold, as measured by quantitative RT-PCR (Figure 10A), resulted in a ∼4 fold statistically significant (p<0.0001) reduction in YFV titer (Figure 10B, compare vector cells treated with the control and DNAJC14 siRNAs). Western blot analysis using anti-DNAJC14 antibody demonstrates a reduction upon silencing at the protein level as well (Figure 10C). Although no protein band is apparent in the vector cells treated with the DNAJC14-targeting siRNA, given that mRNA was still detectable, a low level of residual protein could account for the modest reduction in viral replication. Despite multiple attempts we were unable to reduce the DNAJC14 mRNA levels lower than ∼2 fold (data not shown). Interestingly, cells transduced with lentivirus expressing DNAJC14 had a >300 fold increase in the level of DNAJC14 mRNA (Figure 10A,) and a corresponding increase in DNAJC14 protein levels (Figure 10C), which resulted in a ∼15 fold inhibition of YFV virion production (Figure 10B, compare vector and DNAJC14 cells treated with the control siRNA). Silencing of DNAJC14 in the DNAJC14-overexpressing cells resulted in intermediate mRNA (Figure 10A) and protein (Figure 10C) levels, although the RNA levels remained ∼50–60 fold higher than endogenous levels (compare DNAJC14 siRNA-treated DNAJC14 cells to control siRNA-treated vector cells). This residual intermediate level of DNAJC14 was less inhibitory than the high levels present in DNAJC14 overexpressing cells treated with the control siRNA, but still resulted in a 4.7 fold inhibition of YFV replication compared to vector cells treated with the control siRNA (Figure 10B). Thus decreasing DNAJC14 levels by ∼2 fold or increasing levels by ∼50 fold each had a similar (∼4 fold) inhibitory effect towards YFV. Thus maximal YFV replication requires an optimal DNAJC14 concentration; levels too low or too high result in inhibition.

### The antiviral activity of DNAJC14 occurs in a temporal and dose-dependent manner

A requirement for an optimal level of DNAJC14 and the ability of overexpressed wildtype DNAJC14 to inhibit YFV replication could be explained by DNAJC14 facilitating YFV replication complex formation in a stoichiometric process such that increased levels might function in a dominant negative fashion to inhibit replication complex formation. There is precedent for this, since DNAJC14 modulates BVDV NS2-3 cleavage in a temporal manner due to a stoichiometric mechanism [40], [41]. After translation of the BVDV polyprotein, NS2-3 autoprocessing is mediated in cis by the cysteine protease residing in NS2 [41], which requires DNAJC14 in a 1∶1 ratio [40]. RNA replication is dependent on this cleavage, due to a requirement for free NS3 for the formation of functional replication complexes [41]. Limiting amounts of cellular DNAJC14 thus limit processing and result in downregulation of RNA replication at later time points, allowing viral persistence [40]. Overexpression of DNAJC14 results in increased cleavage at NS2/3, increased RNA replication, and increased cytopathogenicity [39].

**Figure 11:**
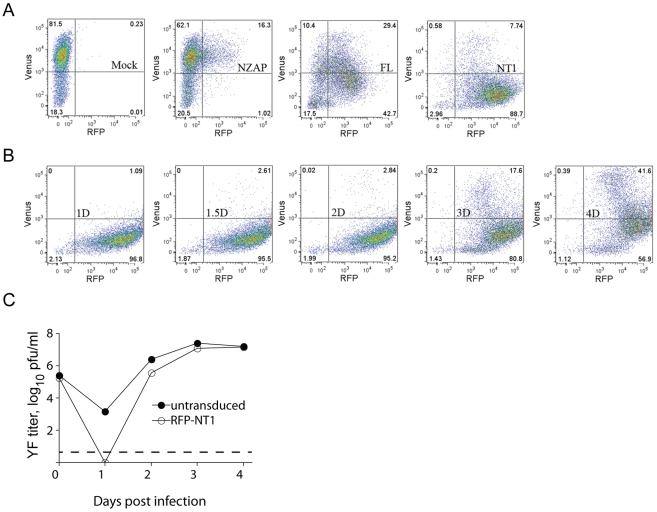
DNAJC14 inhibits YFV in a temporal and dose-dependent manner. (A) SW13 cells were mock transduced (Mock) or transduced with Trip-RFP-hNZAP (NZAP), Trip-RFP-hDNAJC14-FL (FL) or Trip-RFP-hDNAJC14-NT1 (NT1) and infected 2 d later with YFV-Venus (moi = 5). Cells were fixed at 1.5 d post infection and analyzed by flow cytometry. The Venus (y-axis) and RFP (x-axis) fluorescence intensities of the cells are shown; gates to indicate expression of the transduced protein or productive infection were set on Mock transduced, uninfected cells (not shown). (B) SW13 cells were transduced with Trip-RFP-hDNAJC14-NT1 and infected 2 d later with YFV-Venus (moi = 5) and analyzed as in (A) at the indicated days after infection. (C) SW13 cells were left untransduced (closed circles) or were transduced with Trip-RFP-hDNAJC14-NT1 (RFP-NT1, open circles) and infected 2 d later with YFV-Venus (moi = 5). At the indicated times, the medium was removed and YFV present in the medium was quantified by plaque assay. Each data point represents the mean titer obtained from duplicate wells; error bars indicating the range are obscured by the symbols. The dotted line indicates the sensitivity of the plaque assay.

We wondered if inhibition of YFV might exhibit similar properties, in which the ratio of DNAJC14 to viral substrate is critical for its antiviral activity. If so, then inhibition would be expected to be dose-dependent, and continued translation of the incoming genome over time might restore the appropriate stoichiometry and thus allow replication to begin. Consistent with this hypothesis, we noticed that the antiviral activity of DNAJC14 diminished at later times after infection or electroporation (Figures. 4A, 5B). To test this hypothesis, we monitored the antiviral activity of DNAJC14 at the single cell level by flow cytometry. Cells transduced with RFP-tagged DNAJC14 (full-length and NT1 mutant) were infected with a YFV variant expressing Venus, and both virus replication (Venus) and DNAJC14 expression (RFP) were monitored. As shown in Figure 11A, the YFV signal was dramatically reduced in RFP-DNAJC14-FL- and -NT1-expressing cells compared to levels seen in cells expressing ZAP, an anti-Sindbis virus protein with no effect on YFV [10], [51]. In addition, the NT1 mutant demonstrated more potent inhibitory activity than full-length DNAJC14, which may be due to higher expression levels of NT1 as reflected by the RFP signal. Interestingly, both full-length and the NT1 mutant inhibited YFV in a dose dependent manner (Figure 11A), with lower YFV (Venus) signal seen in cells expressing higher levels of DNAJC14 (RFP). We next investigated the antiviral activity of mutant NT1 at various time points after infection (Figure 10B). At late time points (4 d), substantial YFV (Venus) signal was detected in RFP-positive cells. Even at this late time, when substantial YFV replication was occurring, the inhibition mediated by NT1 was still dose-dependent. To verify that increased Venus expression was due to increased replication, in a separate experiment we monitored infectious virus production in cells expressing RFP-DNAJC14-NT1 compared to nontransduced cells. Virion production on day 4 was found to be equivalent (Figure 10C), despite the fact that the DNAJC14 overexpressing cells were more resistant to cell death as monitored by crystal violet staining (data not shown). To exclude the possibility that the increase in virus replication seen after 3 to 4 d is due to the generation and replication of escape mutants, we collected the culture medium from cells after 4 d of infection and re-infected new cells expressing RFP-DNAJC14-NT1. Infection with this virus resulted in a similar early inhibition with a time-dependent increase in virus replication (data not shown). These results demonstrate that not only is DNAJC14-mediated inhibition dependent on the level of DNAJC14, but with time, levels that initially blocked YFV replication no longer are inhibitory. The failure to observe YFV replication in the Rd 3 cells (Figure 3A) is likely due to the fact that these cells had undergone prior infection and selection, resulting in a population of cells with maximal inhibitory properties.

## Discussion

DNAJC14 (also designated DRIP78, Jiv and HDJ3) is a member of the Hsp40 family of protein chaperones [45], [52]. Proteins in this family contain a 70 amino acid motif, designated the J-domain, which recruits Hsp70 family members and stimulates the ATP hydrolysis step of the chaperone process. J-domain containing proteins are involved in diverse cellular processes. The human DNAJC family has 23 members with the presence of the J domain being the single common feature. Although not extensively studied, involvement of these proteins in mitochondrial import, translation, endocytosis and exocytosis has been noted [45]. DNAJC14 has previously been implicated in the life cycle of a member of the *Flaviviridae.* The bovine homolog of this factor, Jiv, is essential for the polyprotein cleavage and replication of the pestivirus BVDV. Jiv acts as a required co-factor for the viral NS2 autoprotease, influencing its cleavage from NS3 and modulating RNA replication, virus production and cytopathogenicity of this pestivirus [38], [39], [42]. In contrast to our findings with YFV, increased expression of Jiv results in higher levels of BVDV RNA replication and virus-induced cell death. Interestingly, some cytopathic biotypes of BVDV are naturally occurring recombinant viruses, which have insertions of DNAJC14 in the NS2-3 coding region. A 90 amino acid domain common to all of the Jiv-containing cytopathic BVDV isolates is designated Jiv90 (see Figure 2D). This sequence is distinct from the J-domain and contains two conserved CXXCXXXH motifs.

DNAJ proteins regulate the ATPase cycle of Hsp70 via their J domain, with the HPD motif critical in accelerating the Hsp70 ATPase activity, while the substrate-binding domain loads the substrate onto Hsp70 [53], [54]. Our studies with mutant H471Q suggest that the critical HPD motif within the J domain is required for DNAJC14 antiviral function, suggesting that ATP-driven Hsp70 chaperone activity may be involved in the process of RNA replication and its inhibition. Since Hsp70 chaperone activity occurs via a stoichiometric mechanism, with a single Hsp70 monomer per substrate [54] it seems likely that DNAJC14/Hsp70 chaperone activity is required for YFV replication complex assembly and that overexpression of DNAJC14 disrupts the chaperone/substrate complex. Dimerization of some Hsp40 family members is evolutionarily conserved and required for their function [45], [53]. The CT1 mutant lacks the ability to multimerize (Figure 8) and fails to inhibit YFV (Figure 6) as well as HCV (data not shown). Thus multimerization is likely critical for DNAJC14's antiviral function.

In our studies, we demonstrated that DNAJC14 noninhibitory mutants are found in YFV replication complexes, as measured by colocalization and coprecipitation with NS3 (Figure 10A and B). Moreover, endogenous DNAJC14 rearranges upon YFV infection and is found colocalized with active replication complexes, as determined by the presence of dsRNA (Figure 10C). This suggests that YFV replication complexes assemble at a specific ER membrane site where DNAJC14 is located, and that DNAJC14 (and likely Hsp70) is specifically recruited to facilitate formation of the viral replication complex. DNAJC14 overexpression would then result in disrupted chaperone/substrate stoichiometry and inhibit replication complex assembly. Alternatively, YFV may hijack DNAJC14-containing membranes for its replication complex assembly, and overexpression may inhibit the distribution and recruitment of other host factors localized to this membrane microdomain and required for replication complex formation.

We realized that the inhibitory effect of DNAJC14 on YFV was diminished at later time points post infection and that inhibition was dose dependent, with higher levels of DNAJC14 resulting in lower levels of virus replication (Figure 11). One possible explanation is that at early time points, overexpressed DNAJC14 is in vast excess to its substrates (viral proteins) and this inappropriate stoichiometry results in inhibition of replication complex formation. Since DNAJC14 does not inhibit virus genome translation (Figure 5D), nor polyprotein processing (Figure 8C), viral protein would be predicted to accumulate with time. At some point, the level of viral protein(s) would result in an appropriate DNAJC14 to substrate ratio to allow the chaperone process to occur and thus overcome DNAJC14's inhibitory effect. This is not dissimilar to the scenario occurring with BVDV, in which the DNAJC14 Jiv90 domain interacts with BVDV NS2-3 at a ratio of 1∶1. This stoichiometric mechanism might be a common requirement for normal DNAJC14 cellular function, as either overexpression or sequestration of DNAJC14 inhibits dopamine D1 receptor transport [44].

We found that multiple *Flaviviridae* were inhibited under conditions of DNAJC14 overexpression and wondered whether viruses from other families might be similarly affected. We tested DNAJC14's effects on Sindbis virus, a positive strand RNA virus from the *Alphavirus* genus. In contrast to the flaviviruses, we found that DNAJC14 overexpression had no effect on viral replication, as measured by expression of a fluorescent reporter from the viral subgenomic RNA (data not shown). Thus replication complex formation for Sindbis virus is not likely affected by DNAJC14 overexpression. Interestingly, however, Sindbis virion production was reduced by DNAJC14 overexpression (data not shown). Thus a step in Sindbis virus assembly, such as glycoprotein maturation and transport from the ER-Golgi to the plasma membrane, where Sindbis budding occurs, may require DNAJC14-containing membrane microdomains and chaperone function. In addition, overexpression of DNAJC14 reduced VSV virion production (data not shown), suggesting an effect on VSV glycoprotein ER-golgi transport. Since it has been reported that DNAJC14 is involved in dopamine D1 receptor transport [44], it is likely that DNAJC14 facilitates specific membrane processes including vesicle transport and viral replication complex assembly. It is possible that many virus families have specific requirements for chaperone processes at various steps in their life cycle. Understanding these requirements and identifying the chaperones and proteins undergoing the chaperone process may lead to insights into similarities and differences between different virus families in these critical life cycle steps.

**Figure 12:**
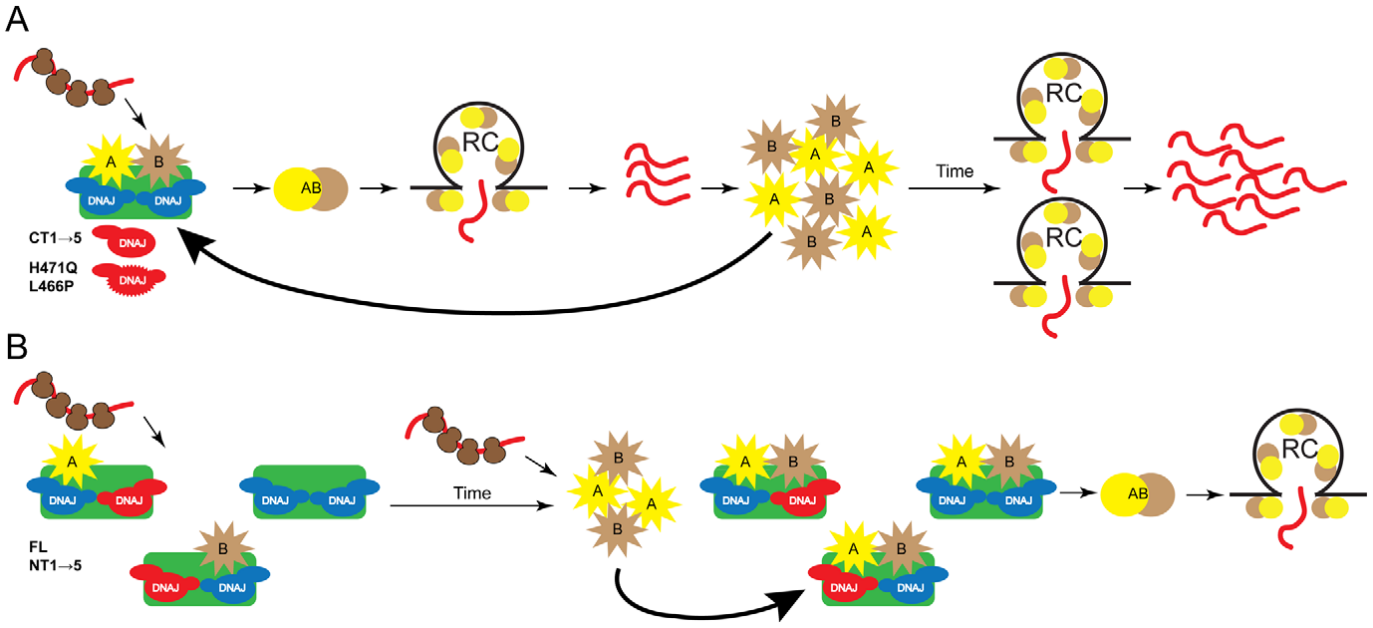
Proposed model of DNAJC14 function. (A) Incoming viral RNA is translated to produce the inactive form(s) of a viral protein(s) required for RNA replication complex formation (A and B, yellow and brown stars). When the stoichiometry of the substrate protein(s) and the host chaperone machinery, consisting of DNAJC14 (DNAJ, blue) and Hsp70 (green), is appropriate, proper folding (AB) allows the formation of replication complexes which generate new progeny viral RNA. This RNA is further translated to produce more substrate, which after undergoing the chaperone process results in the formation of additional replication complexes and amplification of the RNA replication process. Overexpression of DNAJC14 mutants (red) lacking the C-terminal self-interaction domain (CT mutants), or with mutations in the J domain (H471Q, L466P) has no effect on viral replication, since the mutants lack features necessary for stable interaction and the chaperone complex is not disrupted. (B) Overexpression of full-length DNAJC14 (FL) or N-terminal truncation mutants (NT mutants) results in their incorporation into the chaperone complexes due to the presence of the C-terminal interaction domain and an intact J domain. Disruption of the normal stoichiometry of the substrate/chaperone complex results in a failure to properly fold the viral protein and a failure to generate replication complexes. With time, however, continued translation of the incoming genome results in increased concentrations of the viral protein. The appropriate stoichiometry is restored allowing the chaperone process to proceed and viral replication complexes to be generated.

DNAJC14 can both facilitate and inhibit YFV replication. Based on all of our findings, we propose the following model (Figure 12): Translation of the incoming YFV RNA and subsequent polyprotein processing generates the viral proteins necessary for the viral RNA replication process. DNAJC14 functions as a chaperone system, most likely with involvement of Hsp70, to facilitate a step in the YFV membrane-associated multiprotein complex assembly that is critical for the formation of replication complexes. The TM domains within DNAJC14 target the protein to a specific subcellular ER membrane location, wherein substrate selection and YFV replication complex formation occurs. Multimerization of DNAJC14 via its C-terminus is likely required for assembly of the chaperone/substrate complex. It is possible that each DNAJC14 monomer binds a substrate and together they promote the proper folding and interaction of the substrate pair, which might be different sites on the same protein, two different YFV proteins, or a viral protein and host protein necessary for viral replication. Newly generated viral RNA is produced, which after translation generates new substrate for the chaperone process, and the formation of additional replication complexes. Overexpression of DNAJC14 mutants that fail to multimerize (CT1, CT2, CT3, CT4 or CT5), or contain mutations in the critical J domain (L466P, H471Q, Figure 12A) has no effect on virus replication; these mutants fail to interact with and disrupt the normal chaperone components and therefore exhibit no antiviral activity. Expression of full-length (wildtype) DNAJC14 results in an excess of DNAJC14 relative to the substrate, and complexes with an inappropriate stoichiometric ratio are formed, disrupting the chaperone process (Figure 12 B). The N-terminal truncation mutants, which contain the C terminal multimerization motif and an intact J domain, also interact with the chaperone components, disrupting the proper chaperone/substrate stoichiometry. With time, continued translation of the incoming viral genome (or genome generated by very low levels of viral replication) results in the accumulation of viral proteins. Once the optimal substrate/chaperone ratio is established, the restored chaperone process results in replication complex formation and viral RNA replication. Further studies are required to address the viral and cellular substrate(s) for DNAJC14 and to determine if other host factors (for example, Hsp70) participate in this important chaperone process.
